# Telomere-to-telomere and gap-free genome assembly of a susceptible grapevine species (Thompson Seedless) to facilitate grape functional genomics

**DOI:** 10.1093/hr/uhad260

**Published:** 2023-12-13

**Authors:** Xianhang Wang, Mingxing Tu, Ya Wang, Yali Zhang, Wuchen Yin, Jinghao Fang, Min Gao, Zhi Li, Wei Zhan, Yulin Fang, Junyang Song, Zhumei Xi, Xiping Wang

**Affiliations:** College of Enology, College of Food Science and Engineering, Viti-Viniculture Engineering Technology Center of State Forestry and Grassland Administration, Shaanxi Engineering Research Center for Viti-Viniculture, Heyang Viti-Viniculture Station, Northwest A&F University, Yangling, Shaanxi 712100, China; College of Enology, College of Food Science and Engineering, Viti-Viniculture Engineering Technology Center of State Forestry and Grassland Administration, Shaanxi Engineering Research Center for Viti-Viniculture, Heyang Viti-Viniculture Station, Northwest A&F University, Yangling, Shaanxi 712100, China; State Key Laboratory of Crop Stress Biology in Arid Areas, College of Horticulture, Northwest A&F University, Yangling, Shaanxi 712100, China; College of Enology, College of Food Science and Engineering, Viti-Viniculture Engineering Technology Center of State Forestry and Grassland Administration, Shaanxi Engineering Research Center for Viti-Viniculture, Heyang Viti-Viniculture Station, Northwest A&F University, Yangling, Shaanxi 712100, China; State Key Laboratory of Crop Stress Biology in Arid Areas, College of Horticulture, Northwest A&F University, Yangling, Shaanxi 712100, China; State Key Laboratory of Crop Stress Biology in Arid Areas, College of Horticulture, Northwest A&F University, Yangling, Shaanxi 712100, China; State Key Laboratory of Crop Stress Biology in Arid Areas, College of Horticulture, Northwest A&F University, Yangling, Shaanxi 712100, China; State Key Laboratory of Crop Stress Biology in Arid Areas, College of Horticulture, Northwest A&F University, Yangling, Shaanxi 712100, China; Xi'an Haorui Genomics Technology Co., Ltd, Xi'an 710116, China; College of Enology, College of Food Science and Engineering, Viti-Viniculture Engineering Technology Center of State Forestry and Grassland Administration, Shaanxi Engineering Research Center for Viti-Viniculture, Heyang Viti-Viniculture Station, Northwest A&F University, Yangling, Shaanxi 712100, China; College of Landscape Architecture and Arts, Northwest A&F University, Yangling, Shaanxi 712100, China; College of Enology, College of Food Science and Engineering, Viti-Viniculture Engineering Technology Center of State Forestry and Grassland Administration, Shaanxi Engineering Research Center for Viti-Viniculture, Heyang Viti-Viniculture Station, Northwest A&F University, Yangling, Shaanxi 712100, China; State Key Laboratory of Crop Stress Biology in Arid Areas, College of Horticulture, Northwest A&F University, Yangling, Shaanxi 712100, China

## Abstract

Grapes are globally recognized as economically significant fruit trees. Among grape varieties, Thompson Seedless holds paramount influence for fresh consumption and for extensive applications in winemaking, drying, and juicing. This variety is one of the most efficient genotypes for grape genetic modification. However, the lack of a high-quality genome has impeded effective breeding efforts. Here, we present the high-quality reference genome of Thompson Seedless with all 19 chromosomes represented as 19 contiguous sequences (N50 = 27.1 Mb) with zero gaps and prediction of all telomeres and centromeres. Compared with the previous assembly (TSv1 version), the new assembly incorporates an additional 31.5 Mb of high-quality sequenced data with annotation of a total of 30 397 protein-coding genes. We also performed a meticulous analysis to identify nucleotide-binding leucine-rich repeat genes (NLRs) in Thompson Seedless and two wild grape varieties renowned for their disease resistance. Our analysis revealed a significant reduction in the number of two types of NLRs, TIR-NB-LRR (TNL) and CC-NB-LRR (CNL), in Thompson Seedless, which may have led to its sensitivity to many fungal diseases, such as powdery mildew, and an increase in the number of a third type, RPW8 (resistance to powdery mildew 8)-NB-LRR (RNL). Subsequently, transcriptome analysis showed significant enrichment of NLRs during powdery mildew infection, emphasizing the pivotal role of these elements in grapevine’s defense against powdery mildew. The successful assembly of a high-quality Thompson Seedless reference genome significantly contributes to grape genomics research, providing insight into the importance of seedlessness, disease resistance, and color traits, and these data can be used to facilitate grape molecular breeding efforts.

## Introduction

High-accuracy gapless genomes are more informative and can greatly facilitate molecular breeding and gene characterization. With the development of DNA sequencing technology, long-read sequencing technologies, such as Pacific Biosciences (PacBio) single-molecule real-time (SMRT) sequencing and Oxford Nanopore Technology (ONT) sequencing have fundamentally altered approaches to genome assembly [1, 2]. These technologies can generate single molecular reads in excess of 10 kb, spanning most simple repeat sequences in many genomes, making it possible to achieve contiguous and gapless genome assemblies [2]. Moreover, recently developed technologies such as circular consensus sequencing (CCS), which generates high-fidelity (HiFi) sequence reads, optical mapping (Bionano) and chromosome conformation capture sequencing (Hi-C) have further improved the continuity of the assembly [1]. The strategy of combining long-read sequencing (PacBio, ONT), Hi-C, and optical mapping not only reveals the organization and architecture of chromosomes, including complex regions such as centromeres, telomeres, transposable elements (TEs), and segmental duplications [1, 3], but also allows access to the gene catalog of some species [4].

Very recently, multiple telomere-to-telomere (T2T) sequence assemblies were reported for several plant species, including *Arabidopsis thaliana* [2], rice (*Oryza sativa*) [1, 5], barley (*Hordeum vulgare* L.) [3], banana [6–8], maize [9], tea tree (DASZ) [10], tomato (*Solanum lycopersicum*) [11], watermelon (*Citrullus lanatus*) [12], bitter melon (*Momordica charantia* L. var. *abbreviata* Ser.) [13], kiwifruit [14], *Brassica rapa* [15], lemon [*Citrus limon* (L.) Burm. f.] [16], and strawberry (*Fragaria vesca*) [17]. Liu *et al*. [9] used a combination of long-read sequencing (PacBio), ultra-long nanopore (ONT) sequencing, and an optical map to improve the contiguity of the maize genome, revealing an internal structure of seven centromeres and five heterochromatic knob regions. Zhang *et al*. [10] assembled and annotated the genome of an ancient tea tree using SMRT sequencing (PacBio), Hi-C, and RNA sequencing data from an additional 217 tea accessions to clarify the pedigree of tea cultivars and identify some key genes affecting flavonoid biosynthesis in Chinese tea. In a study of an inbred tobacco mosaic virus (TMV)-resistant tomato variety, van Rengs *et al*. [11] applied PacBio HiFi and ONT sequencing to develop highly contiguous and complementary genome assemblies containing all 12 chromosomes that revealed a complex series of structural variants linked to the TMV resistance gene [11]. In rice, Li *et al*. [1] completed gapless assembly of all chromosomes in rice (Minghui 63) using PacBio HiFi, genetic maps, Hi-C, and alignment with genome sequences of related species, finding the TEs and segmental duplications that result in gene duplications necessary for the adaptive evolution of disease resistance and developmental processes. Also studying rice, Song *et al*. [5] assembled two gapless rice (Zhenshan 97 and Minghui 63) genomes and found that centromeric regions share conserved centromere-specific satellite motifs with different copy numbers and structures. In lemon, Bao *et al*. [16] applied PacBio HiFi, ONT, and Hi-C technologies to generate a gap-free and haplotype-resolved lemon genome and identified candidate genes associated with flavor compound biosynthesis and huanglongbing disease tolerance. Collectively, the availability of these gapless genomic data has greatly expanded understanding of plant genome structure and function and the breeding of resistant varieties.

Grapevines (*Vitis* spp.) used for wine, table grapes, and raisins are widely cultivated and the most valuable fruit crops in the world [18, 19]. Since 2007, biological studies in grapes have been greatly facilitated by the assemblies of *Vitis vinifera* (Pinot Noir, PN40024) variety based on Sanger sequencing [20]. Subsequently, additional grape genome sequences of different cultivars have been published, including genomes of *Vitis labruscana* × *V. vinifera* (Shine Muscat) [19], *V. riparia* Michx. (Manitoba 37) [18], *V. vinifera* (Chardonnay, Cabernet Sauvignon, and Sultanina/Thompson Seedless) [21–24], *Muscadinia rotundifolia* (Noble) [25], and *V. amurensis* (Shanputao) [26]. However, most published genome sequences are short-read sequencing assemblies, except for those for Chardonnay, Cabernet Sauvignon, Noble, and Shanputao. A contiguous Cabernet Sauvignon genome was assembled by PacBio sequencing reads and the FALCON-Unzip algorithm [21]. Zhou *et al*. [24] combined long-read (PacBio), short-read, and whole-genome methods to assemble the Chardonnay genome and analyze structural variants. Very recently, contiguous Noble and Shanputao reference assemblies were completed using a combination of PacBio reads, Hi-C, optical mapping, and genome-wide association study (GWAS) data [25, 26]. Shi *et al*. [27] applied PacBio HiFi long reads to reassemble a T2T gap-free reference genome for a Pinot Noir cultivar (PN40024); repetitive sequences, centromeres, and telomeres were annotated and gene annotations of previous versions were incorporated into PN_T2T. These available high-quality grape cultivar genomes lay a solid foundation for further investigations of evolutionary genomics, analysis of phenotype- and resistance-related traits, and the development of breeding programs.

Thinner pericarps and seedlessness are characteristics selected for table grapes. Currently, Thompson Seedless is one of the most planted cultivars for fresh consumption and raisin production, providing the primary source of seedlessness and playing an important role in table grape breeding [22]. Furthermore, Thompson Seedless is currently the most suitable cultivar to study gene function due to its well-established transgenic system (including overexpression and gene editing) [28–32]. However, the current version of the Thompson Seedless genome was assembled through short-read sequencing and contains gaps and missing sequences, and some complex regions (such as TEs, telomeres, and centromeres) have been either mistakenly assembled or were not even sequenced due to the high enrichment in repetitive elements in these regions [23]. Therefore, obtaining complete and continuous genome data for Thompson Seedless will be extremely beneficial to gene function research and breeding programs.

To address this need, we assembled T2T-level gap-free genome sequences for Thompson Seedless by combining data from PacBio-HiFi, ONT ultra-long, and Hi-C technologies. We obtained a T2T-level assembly that includes all 19 chromosomes as 19 contiguous sequences (N50 = 27.1 Mb) with zero gap. The presence of a comprehensive Thompson Seedless genome without assembly gaps presents an unprecedented opportunity to investigate the regions of telomeres and centromeres. Employing various tools, we successfully identified distinctive genes and protein sequences within these previously unexplored regions. This breakthrough provides insight into the genetic content and biological significance of these once enigmatic areas. Furthermore, we conducted a comprehensive identification of NLRs (nucleotide-binding leucine-rich repeat) genes in Thompson Seedless and performed an analysis of their response to grapevine powdery mildew. This study provides a crucial reference for grape functional genomics research and genetic improvement, particularly for disease resistance breeding.

## Results

### Assembly of a gap-free reference genome for Thompson Seedless

We incorporated multiple sequencing technologies (PacBio HiFi, ONT ultra-long, and Hi-C technologies) to obtain a T2T Thompson Seedless genome. In total, we generated ~60.26 Gb (∼119.4× coverage) of PacBio HiFi reads and 59.59 Gb (∼118.1× coverage) of ONT ultra-long reads by using the PacBio Sequel II and ONT platforms for genome assembly, respectively (Supplementary Data Table S1). Additionally, 64.28 Gb (~127.4× coverage) of Hi-C sequencing data were used to anchor contigs (Supplementary Data Table S1). The N50 length of the HiFi reads was >16.16 kb and that of the ONT reads was >100.28 kb (Supplementary Data Table S1, Supplementary Data Fig. S1). We used *K*-mer to evaluate genomic heterozygosity with Illumina data (Supplementary Data Table S2), and it was estimated at ~1.49% (Supplementary Data Fig. S2).

The high-quality PacBio HiFi reads and ONT ultra-long reads were assembled using hifiasm [33] and NextDenovo (v2.5, https://github.com/Nextomics/NextDenovo), respectively. The obtained HiFi and ONT ultra-long draft genome sizes were 524.99 and 603.65 Mb, with N50 of 25.72 and 9.84 Mb, respectively (Supplementary Data Fig. S3A and B). Subsequently, quality assessment with the Benchmarking Universal Single-Copy Orthologs (BUSCO) program [34] revealed that complete sequences in HiFi and ONT draft genomes accounted for ~98.19 and 98.06% (Supplementary Data Fig. S3C and D). To assess the quality of the two draft genomes, we compared them with Illumina sequencing reads and PacBio HiFi reads (Supplementary Data Tables S1 andS2). We used BWA [35], SAMtools [36], and BCFtools [37] and found that the mapping rates between the HiFi draft genome with Illumina sequencing reads and PacBio HiFi reads were 99.11 and 99.83%, and those between the ONT ultra-long draft genome with Illumina sequencing reads and PacBio HiFi reads were 99.44 and 99.92% (Supplementary Data Table S3), suggesting the high accuracy of our assemblies.

**Figure 1:**
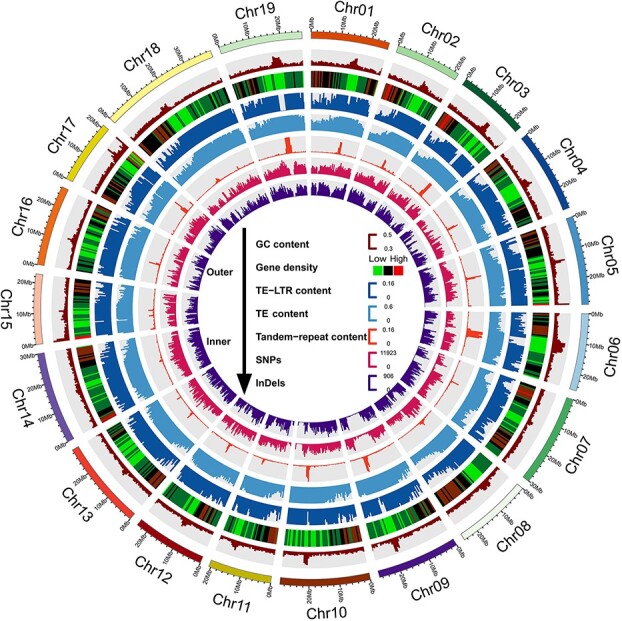
Overview of the T2T and gap-free genome of Thompson Seedless. The Circos plot depicts gene features at 500-kb intervals across the 19 chromosomes of Thompson Seedless. From the outer to the inner ring, the plot shows the GC content, gene density, TE-LTR content, TE content, tandem repeat content, SNPs, and InDels. The WGS reads were mapped to the TSv2_T2T genome for detecting SNPs and InDels. Numbers of SNPs and InDels of Thompson Seedless were calculated per 500 kb. Circos software was used for plotting the figure.

**Figure 2:**
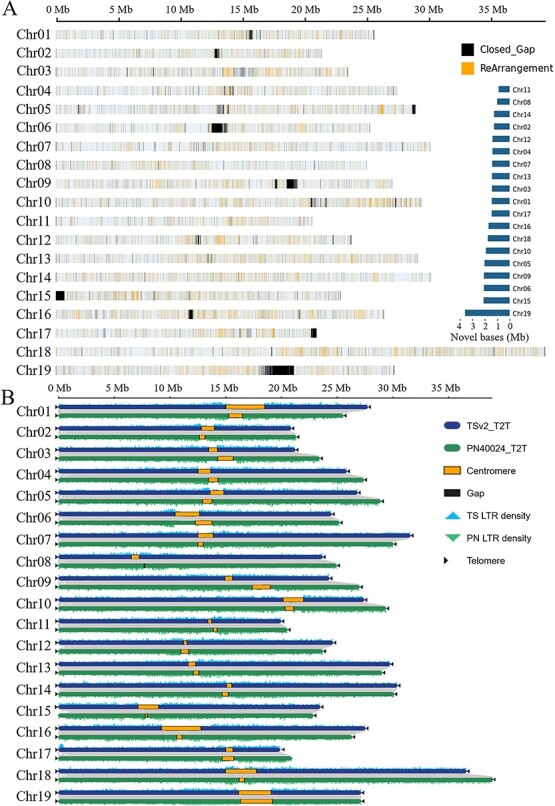
Comparative analysis of the TS_T2T, TSv1, and PN40024_T2T genomes. **A** Genome comparison between TS_T2T and TSv1 versions. MUMmer (v4.0.0) was used to compare the TSv2_T2T with the TSv1 genome using whole-genome alignments. First, nucmer (nucmer —mum) was used to align the two genome sequences and then the variation between chromosomes was analyzed by show-diff. Novel bases represent newly assembled sequences, inserted duplication, and other inserted sequences defined as rearrangements. Numbers represent the size of newly assembled chromosomes on T2T chromosomes. **B** Comparison of TS_T2T with the reference genome PN40024_T2T. Collinear regions are shown by gray lines. Yellow blocks indicate the centromere. Black blocks indicate gap regions. Blue and green triangles indicate LTR density. Black triangles indicate the presence of telomere sequence repeats.

Subsequently, the HiFi draft genome was further grouped, ordered, and orientated into pseudochromosomes using the Hi-C reads. To verify the accuracy of the assisted assembly results, we performed heat map validation on the assembled chromosomes. The global heat map indicated a relatively ideal assembly effect. However, there were strong local interactions between chromosomes, which might reflect clustering errors (Supplementary Data Fig. S4). Although we obtained 9 gap-free chromosomes, this left 10 chromosomes with gaps. In particular, there were four gaps, in chromosome 17 (Supplementary Data Table S4). The ONT contigs were then used to fill the gaps in the HiFi draft genome. After filling all remaining gaps, we achieved a chromosome-level and gap-free genome, designated TSv2_T2T (Fig. 1). The final genome contains 19 chromosomes with a total length of 504.7 Mb, with a contig N50 of 27.1 Mb (Fig. 1, Table 1). The assembly process of the gap-free genome is shown in Supplementary Data Fig. S5. The completeness of the gene repertoire was evaluated using BUSCO. The results showed that ~98.24% of the genes from TSv2_T2T are complete in the assembly, indicating that our TSv2_T2T assembly is high in quality and highly complete (Table 1). Compared with the previous version, TSv1, the new assembled version, TSv2_T2T, filled 15 289 gaps and assembled an additional 31.5 Mb of sequences, including a 3.6-Mb assembly on chromosome 19. Additionally, we reorganized 26 445 previously misassembled blocks (Fig. 2A). The HiFi, ONT, and Hi-C reads were used to assemble two fully separated haplotypes by hifiasm, followed by two-round gap-filling with HiFi and ONT ultra-long reads. Finally, we achieved two gap-free haplotype genomes, Hapl_T2T and Hap2_T2T. Subsequently, we further analyzed and demonstrated the high quality of the two gap-free assemblies (Supplementary Data Table S3, Supplementary Data Fig. S6). For Hapl_T2T, a total of 504.9 Mb sequence length was generated with N50 of 26.3 Mb; for Hap2_T2T, a total of 496.5 Mb sequence length was generated with N50 of 26.5 Mb (Table 1, Supplementary Data Fig. S6).

**Table 1:** Global statistics of Sultanina v2 (*Vitis vinifera*) genome assembly and annotation.

Assembly	TSv2_T2T (diploid)	Hap1_T2T (haplotype)	Hap2_T2T (haplotype)
Assembly length (Mb)	504.7	504.9	496.5
Number of contigs	19	19	19
Contig N50 (Mb)	27.1	26.3	26.5
Longest contig (Mb)	39.4	41.7	38.0
Number of contigs (length ≥2 kb)	19	19	19
Number of gap-free chromosomes	19	19	19
Number of telomeres	38	33	34
Number of definite centromeres	19	19	19
Genome BUSCO (%)	98.24	98.08	98.11
GC content of genome (%)	35.03	34.88	34.97

### Identifying the positions of telomeres and centromeres in chromosomes

Telomeres are basic conserved structures in plant genome sequences that reside at the ends of the chromosomes, and typically consist of short, tandemly arranged minisatellites [38]. Here, referring to the report of Shi *et al*. [27], we predicted all telomeres in the TSv2_T2T genome using the seven-base telomeric repeat (CCCTAAA at the 5′ end or TTTAGGG at the 3′ end) as a sequence query. For the TSv2_T2T genome (diploid), a total of 38 telomeres were predicted (Fig. 2B, Supplementary Data Table S5). The longest telomere was predicted for chromosome 19, 23.7 kb, while the shortest telomere was predicted in chromosome 16, 6.0 kb. For Hapl_T2T (haplotype), 33 out of 38 telomeres were predicted; for Hap2_T2T (haplotype), 34 out of 38 telomeres were predicted (Supplementary Data Tables S6 and S7). For centromeric localization, we scanned candidate repeat sequences using Tandem Repeats Finder (TRF) and identified centromeric sequences in each chromosome of the TSv2_T2T genomic sequence. The longest centromere was in chromosome 19, 2.8 Mb, and the shortest centromere was in chromosome 8, 30 kb (Supplementary Data Table S8).

In the centromeric region of TSv2_T2T, 545 candidate genes were identified. Kyoto Encyclopedia of Genes and Genomes (KEGG) functional enrichment analysis indicated that these genes are mainly related to ‘plant pathogen interaction’, ‘MAPK signaling pathway’, ‘plant hormone signal transduction’, and ‘phenylalanine, tyrosine and tryptophan biosynthesis’ (Supplementary Data Table S8, Supplementary Data Fig. S7). Centromeres were also predicted in all chromosomes of the Hap1_T2T and Hap2_T2T genomes except for chromosome 18 (Supplementary Data Tables S9 and S10).

### Genome annotation analysis

**Figure 3:**
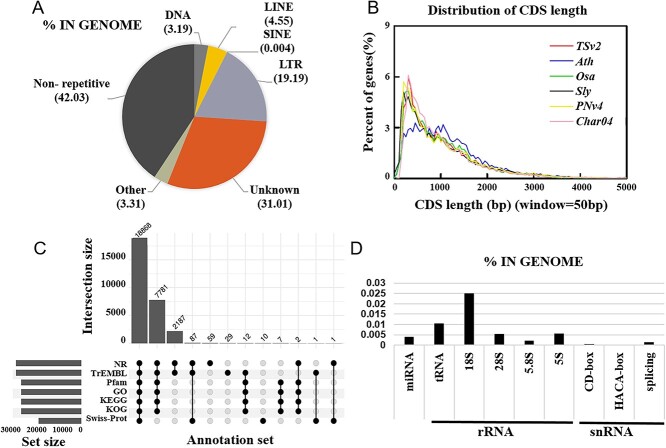
Annotation of the TSv2_T2T genome. **A** TE identification using RepeatMasker and RepeatProteinMask software with RepBase libraries. **B** Distribution of coding sequence (CDS) lengths in TSv2_T2T and other species genomes (*A. thaliana*, *O. sativa*, *S. lycopersicum*, *V. vinifera* – PN40024 and Chardonnay) based on homology-based annotation. **C** Gene function annotation based on various databases. The left bar chart indicates the total number of genes annotated in the same database. The upper panel bar chart represents the number of genes annotated in one or more databases. **D** Annotation of non-coding RNAs. The *x*-axis represents the type of non-coding RNAs and the *y*-axis represents the proportion of the genome.

The repetitive sequences (tandem repeats and TEs, including RNA-mediated TEs) in the TSv2_T2T assembly were annotated by RepeatMasker, RepeatProteinMask, RepeatModeler, and TRF software. Overall, 58.78% of the genome was found to be repetitive (296.69 Mb in total length). Of this repetitive sequence, 7.9% of the genome was identified as tandem repeat sequences through TRF (Supplementary Data Table S11). Analysis revealed 3.19% DNA transposons, 4.55% LINEs (long interspersed nuclear elements), 0.004% SINEs (short interspersed nuclear elements), and 19.19% LTRs (long terminal repeats) in the genome, with total lengths of 16.11, 22.97, 0.02, and 96.86 Mb, respectively (Fig. 3A, Supplementary Data Table S12).

Next, to predict the gene structure from the TSv2_T2T genome, we used several different programs based on different prediction principles. As shown in Supplementary Data Fig. S8, Augustus, SNAP, and GeneMark were separately used for *ab initio* gene prediction, and Blast and Genewise were separately used for homologous prediction. Homology-based annotation was performed using the reference genomes of different species, including *A. thaliana*, *O. sativa*, *S. lycopersicum*, and *V. vinifera* (PN40024 and Chardonnay). In addition, we used PASA and TopHat to predict gene structure by comparing EST/cDNA sequences with genomes. Finally, EvidenceModeler was used to integrate the gene sets predicted by various methods into a non-redundant and more complete gene set. As a result, 30 397 protein-coding genes were predicted in the assembled genome, with an average gene length of 4540.5 bp and an average of 4.6 exons per gene (Supplementary Data Table S13). We compared and analyzed the distribution of gene composition characteristics between the assembled TSv2_T2T genome and the reference genomes of *A. thaliana*, *O. sativa*, *S. lycopersicum*, and *V. vinifera* (PN40024 and Chardonnay), and found relatively similar average coding sequence length (Fig. 3B).

We used the KEGG, Gene Ontology (GO), Pfam, non-redundant (NR), COG/KOG, Swiss-Pro and TrEMBL databases for gene function annotation (Fig. 3C). A total of 29 044 (95.65%) genes were functionally annotated from these databases. Of these genes, 18 868 (62.14%) were functionally annotated in all searched databases, with 26 670 (87.83%) genes annotated in the COG/KOG database, 13 232 (43.58%) genes annotated in the KEGG database, 12 938 (42.61%) genes annotated in the GO database, 24 113 (79.41%) genes annotated in the Pfam database, 28 985 (95.46%) genes annotated in the NR database, 18 967 (62.46%) genes annotated in the Swiss-Prot database, and 28 965 (95.39%) genes annotated in the TrEMBL database. Simultaneously, we utilized the Rfam and tRNAscan-SE databases to predict non-coding RNAs (ncRNAs). In total, 172 microRNA (miRNA), 704 transfer RNA (tRNA), 978 ribosomal RNA (rRNA), and 83 small nucleolar RNA (snRNA) genes were identified in the TSv2_T2T genome (Supplementary Data Table S14).

### Comparative genomic analysis

**Figure 4:**
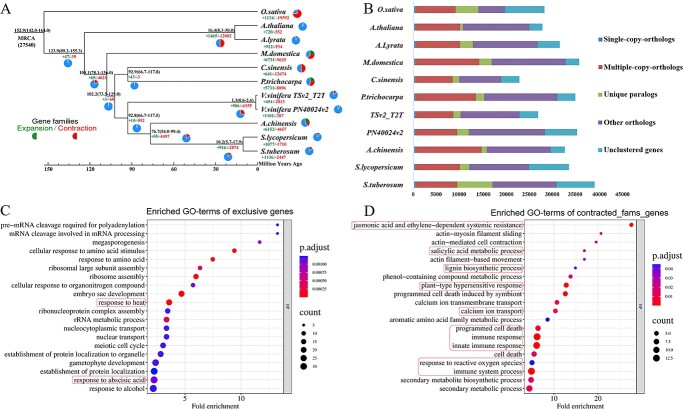
Comparative phylogenetic and collinearity analysis of Thompson Seedless (TSv2_T2T) and related plant species. **A** Phylogenomic analysis and gene family distribution of Thompson Seedless in comparison with 10 other plant species. Divergence time from the present (in million years ago, MYA) is indicated on the nodes. Red and green numbers indicate contracted and expanded ortholog groups. **B** Gene distribution of all gene families across the 11 plant species, including Thompson Seedless. Different types of orthologous and paralogous gene families are distinguished by different colors. **C**, **D** GO functional enrichment analysis of exclusive genes (**C**) and contracted family genes (**D**) observed in Thompson Seedless. The *y*- and *x*-axis labels represent the pathway and fold enrichment, respectively. Size and color of the bubbles represent numbers of genes enriched and enrichment significance, respectively.

Using OrthoFinder [39], we compared the TSv2_T2T genome with the genomes of 10 other plant species to analyze family genes and the dynamics of gene family expansion and contraction (Fig. 4A). For the TSv2_T2T genome, 26 554 (87.45%) genes were categorized into 23 068 gene families. Among them, 1205 exclusive genes and 3486 unclustered genes were identified only in the TSv2_T2T genome (Supplementary Data Tables S15 and S16). A total of 501 common single-copy families were identified, and the family number distributions for the 11 analyzed species are shown in Fig. 4B. Noticeably, multiple-copy families were lower in number in the TSv2_T2T genome than in the other 10 genomes (Fig. 4B). An evolutionary tree analysis was constructed based on the set of 501 single-copy family genes, with the outgroup represented by *O. sativa*. The result indicated that Thomson Seedless and PN40024 shared a common ancestor ~1.3 (0.6–2.6) million years ago. Further analysis indicated that 567 genes were expanded and 225 were contracted in TSv2_T2T (Fig. 4A).

GO functional enrichment analysis based on clusterProfiler [40] revealed that the exclusive genes predicted in the TSv2_T2T genome were mainly enriched in processes of ‘establishment of protein localization’, ‘gametophyte development’, ‘rRNA metabolic process’, and ‘embryo sac development’. In addition, many enriched GO terms were associated with plant response to stimuli, such as ‘response to alcohol’, ‘response to abscisic acid’, and ‘response to heat’ (Fig. 4C, Supplementary Data Table S17). The expanded genes were significantly enriched in the GO terms ‘UDP-glucosyltransferase activity’, ‘quercetin-3-*O*-glucosyltransferase activity’, and ‘quercetin-7-*O*-glucosyltransferase activity’ (Supplementary Data Fig. S9). The contracted genes were significantly enriched in the GO terms ‘response to reactive oxygen species’, ‘lignin biosynthetic process’, ‘salicylic acid metabolic process’, ‘cell death’, and ‘immune response’, all terms associated with plant–pathogen interaction (Fig. 4D, Supplementary Data Table S17).

### Genome structural variation

**Figure 5:**
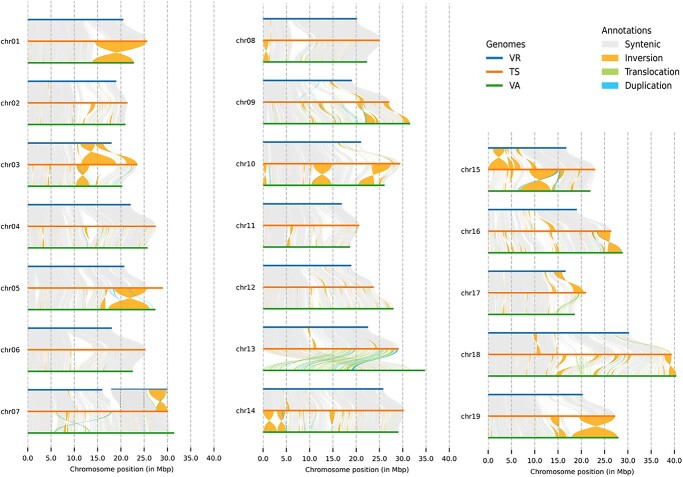
Comparative analysis of genome synteny and rearrangement. Synteny and rearrangement (SyRI) plot depicting chromosome comparison among *V. amurensis* (VA), diploid Thompson Seedless genome (TSv2_T2T), and *M. rotundifolia* cv. Noble (VR). Alignments were constructed using minimap2. SyRI was used to analyze variations. Syntenic regions, inversions, translocations, and duplications are highlighted by different colors.

**Figure 6:**
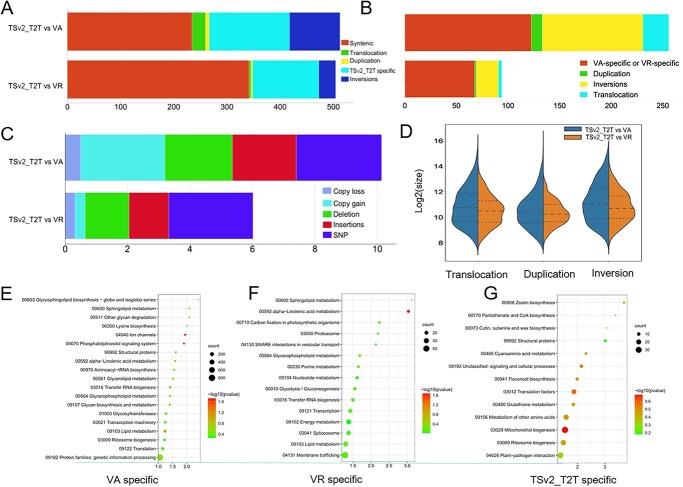
Comparative analysis of structural variations between TSv2_T2T, VA, and VR. Structural variations were evaluated by two comparisons: TSv2_T2T versus VR with VR as the reference genome and TSv2_T2T as the query genome, and TSv2_T2T versus VA with TSv2_T2T as the reference genome and VA as the query genome. **A**, **B** Bar plots illustrating the total length of structural variations. **C** Bar plot depicting sequence differences in structural variations for TSv2_T2T versus VR and TSv2_T2T versus VA. **D** Size distributions of structural variations. **E**–**G** KEGG functional enrichment analysis of genes in VA-specific (**E**), VR-specific (**F**), and TSv2_T2T-specific (**G**) chromosomal regions. The *y*- and *x*-axis labels represent pathway and enrichment factor, respectively. Size and color of the bubbles represent numbers of genes enriched and enrichment significance, respectively.

We next conducted comparative analysis of the genomes of Thomson Seedless and two wild *Vitis* species, *V. amurensis* [26] and *M. rotundifolia* cv. Noble [25]. The goal of this comparison was to identify structural variations, including duplications, inversions, and translocations, and sequence differences such as deletions and insertions (Figs 5 and6). The genome comparison revealed a high degree of synteny among the three cultivars, as shown in Figs 5 and6A. The syntenic regions accounted for ~341.5 Mb in the TSv2_T2T versus VR (*M. rotundifolia* cv. Noble) comparison and 234.6 Mb in the TSv2_T2T versus VA (*V. amurensis*) comparison. Translocation was ~3.3 Mb for TSv2_T2T versus VR and 24.9 Mb for TSv2_T2T versus VA (Fig. 6A). We observed specific genomic regions ranging from 124.3 to 156.6 Mb that were unique to TSv2_T2T compared with the other two cultivars (Fig. 6A), with 122.6 and 67.6 Mb specific genomic regions for VA and VR, respectively (Fig. 6B). Additionally, we performed KEGG annotation analysis on the genes in the VA-, VR-, and TSv2_T2T-specific regions, and the results are shown in Fig. 6. Both VA- and VR-specific regions were enriched in lipid metabolism, α-linolenic acid metabolism, and glycerophospholipid metabolism. TS-specific regions were enriched in plant–pathogen interaction, glutathione metabolism, and flavonoid biosynthesis (Fig. 6E–G). Most notably, α-linolenic acid metabolism in Fig. 6F and ion channels and phosphatidylinositol signaling system in Fig. 6E are significant (*P*-value <.05, Supplementary Data Table S17). The Hap1_T2T and Hap2_T2T genomes were compared, as shown in Supplementary Data Fig. S10. Approximately 405 Mb syntenic regions, 20 Mb translocations, and 11 Mb inversions were found (Supplementary Data Figs S10 andS11). The ONT and HiFi reads were mapped to Hap1_T2T and Hap2_T2T to prove the accuracy of assembly (Supplementary Data Figs S12 andS13, Supplementary Data Table S18) according to previous research [41].

### Identification of nucleotide-binding leucine-rich repeat genes

A class of NLR genes containing conserved NB-ARC core functional domains and diverse amino (CC or TIR) domains and carboxyl (LRR) domains can regulate plant disease resistance and immune processes [42]. The products of these genes directly or indirectly recognize effector factors, induce a strong hypersensitive cell death response, and hinder the ability of pathogens to absorb nutrients, thus limiting them to the infected site and ultimately leading to plant resistance [43]. NLRs include three types: TIR-NB-LRR (TNL), CC-NB-LRR (CNL), and RPW8 (resistance to powdery mildew 8)-NB-LRR (RNL) [44]. Interestingly, we found a large number of NLRs in the contracted gene families of TSv2_T2T.

**Figure 7:**
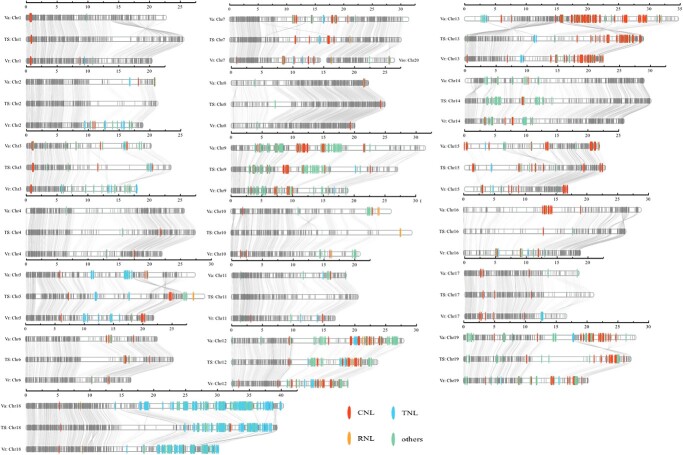
Identification and chromosomal localization of NLR genes in grape species. Chromosomal distribution of NLR genes in *V. amurensis* (VA), diploid Thompson Seedless genome (TSv2_T2T), and *M. rotundifolia* cv. Noble (VR). Classification of NLR genes based on their domain architecture. CNL represents NLR proteins with CC (coiled-coil) domain, RNL represents NLR proteins with RPW (resistance to powdery mildew) domain, TNL represents NLR proteins with Tir (translocated intimin receptor) domain, and ‘others’ represents NLR genes with NB-LRR (nucleotide-binding leucine-rich repeat) domain. OrthoFinder (version 2.5.4) software was used to analyze the single-copy gene families of three grape germplasms, with gray lines representing the gene connections of single-copy gene families.

We identified the NLRs in the genomes of Thomson Seedless and two resistant wild *Vitis* species, *V. amurensis* [26] and *M. rotundifolia* cv. Noble [25]. We identified 107, 184, and 158 TNLs; 244, 356, and 264 CNLs; and 7, 4, and 5 RNLs for Thomson Seedless, *V. amurensis* [26], and *M. rotundifolia* cv. Noble [25], respectively (Fig. 7, Supplementary Data Fig. S14). Compared with the two resistant varieties, Thompson Seedless exhibited reduced numbers of CNL and TNL genes. Specifically, no NLR genes were found in chromosomes 2 and 11 of Thompson Seedless, whereas the wild species had a substantial number of NLR genes in these regions. The deletion of these genes in Thompson Seedless may explain the reduced resistance to various diseases. In contrast to CNL and TNL genes, we observed the opposite trend for RNL genes. Specifically, more RNL genes were detected in the susceptible cultivar Thompson Seedless compared with the two resistant cultivars. In addition, we observed clusters of NLRs in all three grape genomes. The majority of CNL genes were found to be clustered on chromosomes 9, 13, 15, and 17, and TNL genes were predominantly clustered on chromosome 18 (Fig. 7). In contrast, RNL genes were dispersed across all chromosomes without a specific clustering pattern.

### Nucleotide-binding leucine-rich repeats are involved in grape resistance to powdery mildew infection

**Figure 8:**
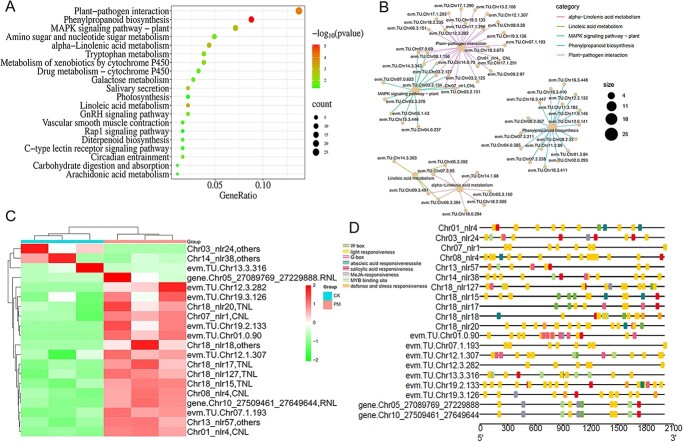
Transcriptome analysis reveals the pivotal role of NLRs in grapevine response to powdery mildew. **A** KEGG functional enrichment analysis of differentially expressed genes at 0 h and 24 h post-inoculation with powdery mildew. The *y*- and *x*-axis labels represent pathway and enrichment factor, respectively. Size and color of the bubbles represent numbers of differentially expressed genes enriched and enrichment significance, respectively. **B** Significantly enriched KEGG pathways and their associated gene networks. Bubble size represents the number of differentially expressed genes enriched. **C** Expression profile of NLR genes in response to powdery mildew. Displayed are all differentially expressed NLRs induced by powdery mildew (log_2_FC > 1 or log_2_FC < −1). **D** Prediction of regulatory elements within the promoter region (2000 bp upstream of the translation start site) of differentially expressed NLR genes. Different colors represent different types of *cis*-acting elements in the promoter.

Powdery mildew is one of the most serious fungal diseases limiting grape cultivation. As a grape variety susceptible to powdery mildew, Thompson Seedless can be used to discover functional genes [45]. Therefore, we performed transcriptome analysis on Thompson Seedless leaves 0 and 24 h after inoculation with powdery mildew, and then used the assembled TSv_T2T as a reference genome for transcriptome analysis (Fig. 8). The analysis yielded mapping rates of transcriptome samples in the range of 93.46–94.88%, with the identification of a total of 698 upregulated genes and 107 downregulated genes.

KEGG enrichment analysis of the differential genes found significant enrichment in plant–pathogen interaction (ko04626), phenylpropanoid biosynthesis (ko00940), linoleic acid metabolism (ko00591), α-linolenic acid metabolism (ko00592), MAPK signaling pathway – plant (ko04016), and other pathways (Fig. 8A). We constructed a network diagram of genes that were significantly enriched in the metabolic pathways of the first five pathways, as shown in Fig. 8B. Of note, most of the genes enriched in the plant–pathogen interaction (ko04626) pathway were NLRs. We identified 20 genes that were significantly differentially expressed by powdery mildew, among which three genes were downregulated and 17 were upregulated, with two RNL genes among the upregulated genes (Fig. 8C).

Subsequently, promoter element analysis was performed using the promoter regions (2000 bp upstream of the translation start site) of all differentially expressed NLR genes (Fig. 8D). We found that hormone response elements for MeJA, abscisic acid, and salicylic acid responsiveness were significantly enriched in the promoter regions, indicating that these hormones and sites are involved in the regulation of NLR-mediated regulation of powdery mildew. In addition, W-box, G-box, and MYB binding sites were also significantly enriched in the promoter regions of NLRs, indicating that WRKY, bZIP, and MYB family members potentially regulate the expression of NLRs and participate in resistance to disease.

## Discussion

Thompson Seedless is a ‘white’ (light green) oval-shaped seedless grapevine variety, also known as ‘Sultana’. Its fruits are used for fresh food, as dried fruit, and for wine, so this variety is widely planted around the world. In recent years, with the development of functional genomics, grape genetic transformation to verify homologous genes has become a routine method [30, 32, 45]. However, there are mature genetic transformation systems for only a few genotypes in grapes, including Thompson Seedless, so many previous studies have used this variety to obtain stable transgenic lines and demonstrated that it has high transformation efficiency [30–32, 45]. Despite its wide use for this purpose, the lack of a high-quality reference genome of Thompson Seedless has limited mutant library preparation of grape using CRISPR/Cas9, due to the difficulty of sequence-specific detection before large-scale sgRNA design, thus affecting prediction of off-target sites. Therefore, the assembly of a high-quality Thompson Seedless reference genome is urgently needed.

In this study, we assembled a 504.7-Mb gap-free and T2T heterozygous genome with an N50 of 27.1 Mb using HiFi, Hi-C, and ONT methods, and identified the telomeres and centromeres on 19 chromosomes. The same approach has been demonstrated for T2T genome assembly in multiple species, such as *A. thaliana* [2], tomato (*Solanum peruvianum*) [11], strawberry (*F. vesca*) [17], kiwifruit (*Actinidia chinensis* cv. Hongyang) [14], and grapevine (*V. vinifera* cv. PN40024) [27]. In 2014, the first version of Thompson’s non-nuclear genome (TSv1) was assembled [22]. Compared with the previously published TSv1 genome, TSv2_T2T assembled an additional 31.5 Mb, with the N50 about 347 times that of the previous genome (Fig. 2A, Table 2), similar to the improvement achieved by recent T2T genome assemblies of other species. Additionally, TSv2_T2T corrected a large number of assembly errors in the TSv1 version and filled a large number of gaps (Fig. 2A). The centromere and telomere regions are rich in heavy sequences, which was one of the main limitations to perfect genome assembly. The genomes of many species lack complete assembly of centromere and telomere regions. In this study, the TSv2_T2T genome assembled 19 centromeres and 38 telomeres, with longest lengths of 2.8 Mb and 23.7 kb, respectively, while PN40024_T2T has only 36 telomeres (Fig. 2B). Second, gap-free assembly was achieved in all 19 chromosomes in the TSv2_T2T genome, further demonstrating the high assembly quality of the TSv2_T2T genome for use as a grapevine reference genome.

**Table 2:** Assembly statistics of Thomson Seedless and other grapevine genomes used in this study.

**Cultivar**	**Abbreviation**	**Assembly statistics**			**Annotation**		
		**Assembly size (Mb)**	**Contig N50 (Mb)**	**Scaffold N50 (Mb)**	**Genes**	**BUSCO (%)**	**TEs (%)**
Sultanina v2 (*V. vinifera*)	TSv2_T2T	504.7	27.1		30 397	98.19	57.97
Sultanina v1 (*V. vinifera*)	TSv1	522.4	0.015	0.071	29 555	93.8	
Pinot Noir derived (*V. vinifera*)	PN40024v2	486	0.102	3.4	28 516	96.9	47.0
PN40024_T2T	494.87	26.9		37 534	98.5	66.47
Noble (*M. rotundifolia*)	VR	393.8	0.11	16.6	26 394	96.0	34.7
Shanputao (*V. amurensis*)	VA	604.56	0.28	0.75	32 885	94.6	47.06

Using the newly assemble genome, we annotated the genes using multiple strategies, and obtained a total of 30 397 protein-coding genes and 57.97% TEs. Previous studies identified 28 516, 26 394, and 32 855 protein-coding genes and 47.0, 34.7, and 47.06% TEs in the genomes of PN40024v2, VR, and VA, respectively (Table 2). Compared with PN40024v2, VR, and VA, more coding genes and repetitive sequences were identified within TSv2_T2T, reflecting the high quality of this genome assembly.

Thompson Seedless originated from the semi-arid Middle East and was introduced into California in 1872 by William Thompson [46]. In China, the main Thompson Seedless producing area is in Xinjiang. Both Xinjiang and California are considered areas with relative water scarcity and heat, which means that Thompson Seedless traditionally grows under a dry climate [47, 48]. In this study, comparative genomic analysis results indicated significant enrichment of exclusive genes in TSv2_T2T in GO terms of ‘response to abscisic acid’ and ‘response to heat’ (Fig. 4C). Numerous studies have shown that the abscisic acid signaling pathway is crucial in regulating plant drought resistance [49–51]. Thus, the presence of these elements likely explains at the genetic level the adaptation of Thompson Seedless to survival in a dry climate.

In general, a positive correlation exists between divergence time and the degree of variation in gene families. It is worth noting that the observed changes in gene families are more substantial between Thompson and PN40024 (diverged ~10 000 years ago) than between * A. thaliana* and *A. lyrata* (which diverged ~12 million years ago; Fig. 4A). These results align with previous studies [52–55], indicating that there may not always be a direct correspondence between divergence time and alterations in gene families. To provide further support for the documented alterations in gene families, we undertook a comparative analysis of intron sizes between the two assemblies TSv2_T2T and PN40024v2. Remarkably, PN40024 showcases a higher prevalence of small introns (Supplementary Data Fig. S15). PN40024 originated as a wine grape variety in Europe, while Thompson Seedless originated from the semi-arid Middle East [46], where it is grown as both a fresh and dried grape variety. The simultaneous emergence of wine and table grapes in different geographic regions led to significant genetic divergences during their early evolution. These distinctions, influenced by artificial selection, likely played a role in the observed variations in gene families within these two germplasms [56–58].

Thompson Seedless is susceptible to various pathogens, including powdery mildew, downy mildew, and *Botrytis cinerea* [30, 45]. Evolutionary tree analysis constructed from the genome of TSv2_T2T and 10 other plant species showed 225 contraction genes in TSv2_T2T. As expected, the functions of these contraction genes were significantly enriched in ‘response to reactive oxygen species’, ‘lignin biosynthetic process’, ‘salicylic acid metabolic process’, ‘cell death’, and ‘immune response’ (Fig. 4D), processes that are considered important immune responses for plant disease resistance [32, 45]. PAMP-triggered immunity (PTI) and effector-triggered immunity (ETI) are two key pathways for plants to resist pathogen invasion. Recently, multiple studies determined that PTI and ETI are not independent immune mechanisms, but instead induce sustained and strong immune responses through mutual reinforcement, with NLRs playing an important regulatory role in these responses [43, 59]. Interestingly, many NLRs were identified in the contracted family genes of TSv2_T2T. In addition, the comparative analysis of susceptible Thomson Seedless and two other resistant wild grape germplasms showed a large number of genomic variations (Figs 5 and6), with fewer NLRs in Thomson Seedless than in the other two varieties. For example, Thomson Seedless lacks prominent NLRs on chromosomes 2 and 11 (Fig. 7). The sensitivity of Thomson Seedless to diverse pathogens is presumably due to the loss of a large number of disease resistance genes, including NLRs, during evolution. Similar phenomena have also been found in other species. For example, Wang *et al*. [60] found large differences in the diversity and abundance of NLRs between disease-resistant and susceptible rice cultivars. Secondly, resistance genes (R genes) exist in clusters, with a large number of tandem repeats [44, 61]. In the comparison of the three grapevine genomes, TNL genes clustered on chromosome 18, and CNL genes mainly clustered on chromosome 13 (Fig. 5, Supplementary Data Table S19).

CNL, TNL, and RNL proteins are pivotal for plant disease resistance. While CNL and TNL proteins recognize and directly respond to pathogen invasions, RNL proteins act as ‘helper’ NLRs downstream of CNL and TNL signaling pathways [44, 62, 63]. The broad-spectrum resistance of RNL proteins to powdery mildew in *Arabidopsis* is well documented. In grapes, the identification of RNLs lagged behind. In 2015, Qiu *et al*. [64] reported the induction of a single grape RNL gene in Thompson Seedless in response to powdery mildew infection. Our transcriptome data expanded this by identifying two additional RNL genes (Fig. 8), confirming the high-quality genome assembly and comprehensive NLR gene annotation in grapes. Wild disease-resistant grape varieties boast a significantly higher number of CNL and TNL genes compared with susceptible Thompson Seedless (Supplementary Data Fig. S14). This suggests that wild grapes can mount quicker and more robust responses to pathogen invasions, enhancing resistance to powdery mildew. This aligns with our previous findings, explaining the rapid activation of numerous disease resistance regulatory genes in disease-resistant grape varieties [65]. Interestingly, susceptible varieties exhibit an increased number of RNL genes. Over time, cultivated grape varieties, such as Thompson Seedless, have faced heightened pathogen threats, notably from powdery mildew. This interaction may have led to a co-evolutionary response. Cultivated varieties expanded their RNL gene repertoire to adapt to severe pathogen pressures, while wild grape varieties inherently possess robust pathogen resistance, experiencing milder pathogen threats. This variation in RNL gene numbers correlates with prior research [66, 67]. Smaller pathogen threats are linked to less pronounced variations in RNL gene numbers. In light of these findings, we propose a hypothesis: wild and cultivated grapes employ divergent strategies against pathogen invasions. Wild grapes prioritize rapid pathogen detection and response, expending significant energy. In contrast, cultivated varieties allocate energy resources to enhance fruit quality, potentially diminishing early pathogen detection. Instead, they bolster downstream disease resistance responses by increasing NLR numbers.

Additionally, KEGG enrichment analysis found that differential genes were mainly enriched in plant–pathogen interaction (ko04626), phenylpropanoid biosynthesis (ko00940), linoleic acid metabolism (ko00591), α-linolenic acid metabolism (ko00592), MAPK signaling pathway – plant (ko04016) (Fig. 8A). Among them, plant–pathogen interaction (ko04626), phenylpropanoid biosynthesis (ko00940), and MAPK signaling pathway – plant (ko04016) are recognized plant disease resistance pathways [32, 59]. Most of the genes enriched in the plant–pathogen interaction (ko04626) pathway are NLRs (Fig. 8), which also shows that NLRs play an important role in grape defense against powdery mildew infection. Subsequently, the analysis of regulatory elements revealed that WRKY, bZIP, and MYB family members may directly regulate the expression of NLRs. We previously found that *VqWRKY52*, *VqWRKY31*, *VqWRKY56*, and *bZIPC22* are all involved in the regulation of grape powdery mildew, and can significantly induce cell death [32, 45, 68]. NLRs can be regulated by the MAPK pathway (PTI enhances ETI), and then regulate resistance to powdery mildew by inducing hypersensitive death [59]. The WRKY family genes may directly regulate the expression of NLR genes to improve resistance to powdery mildew. Interestingly, the α-linolenic acid metabolism (ko00592) pathway was significantly enriched after powdery mildew infection, though this pathway was not previously associated with resistance to powdery mildew. However, this pathway was enriched in both VA-specific and VR-specific genomic regions (Fig. 6E and F), suggesting that future work should evaluate its potential contribution to wild grape disease resistance.

## Materials and methods

### Plant materials

Plant samples were collected from a 5-year-old *V. vinifera* L. cv. Thompson Seedless growing in the grape germplasm resource orchard of Northwest A&F University (Yangling, Shaanxi, China). Thirteen tissues (adult leaves, flowers, fine roots, fruit set, mature fruits, preveraison, ripening seeds, ripening skin/flesh, tendrils, veraison, young leaves, callus, and somatic embryo) were collected from the same plant for sequencing and gene expression analysis.

### Library preparation and sequencing

Genomic DNA was extracted with the Qiagen^®^ Genomic Kit (Cat #13343) according to the instruction manual. DNA purity and concentration were determined using a NanoDrop™ One UV-Vis spectrophotometer (Thermo Fisher Scientific, USA), and Qubit^®^ 3.0 Fluorometer (Invitrogen, USA). For PacBio HiFi sequencing, ~2 μg DNA was used for each SMRTbell library construction according to PacBio’s standard protocol (Pacific Biosciences, CA, USA). The library was then purified and sequenced on a PacBio Sequel II instrument with a Sequel II Sequencing Kit 2.0. Similarly, the ONT library was prepared with 2 μg DNA and sequenced on a Nanopore GridION X5/PromethION sequencer (Oxford Nanopore Technologies, UK). Hi-C library preparation and sequencing were based on a previously described protocol [69].

### Gap-free genome assembly

To obtain a high-quality genome, a variety of different bioinformatics tools were used for *de novo* assembly and gap filling, as shown in Supplementary Data Fig. S5. Briefly, the raw HiFi reads produced were processed using SMRTlink (v10.1.0) (https://github.com/WenchaoLin/SMRT-Link) to remove low-quality reads and adaptors. The high-quality HiFi reads after quality control were used for genome assembly using hifiasm (v0.15.5) with default parameters [33]. The contigs of the draft genome assembled by hifiasm (v0.15.5) were clustered, ordered, and oriented with Hi-C reads to generate a chromosome-scale genome using ALLHiC [70]. The primary assembly was aligned to PN40024v2 with NUCmer (nucmer —mum -D 5), then filtered with a minimum alignment length of 8000 bp (delta-filter -i 85 -l 8000) [27]. Mummerplot was used to visualize collinear relationships between genomes [71] (Supplementary Data Fig. S3B). The chromosome ID and orientation were adjusted manually according to PN40024v2. NextDenovo (v2.5.0, https://github.com/Nextomics/NextDenovo) was used for ONT assembly with the overlap layout-consensus/string graph method, and the contigs were refined with NextPolish (v1.3.1) using Illumina reads with default parameters to improve the accuracy of the assembly [72]. Subsequently, in order to generate a gap-free genome, ONT contig was used to fill the gaps in the genome after Hi-C-assisted assembly using TGS-GapCloser (v1.2.1, https://github.com/BGI-Qingdao/TGS-GapCloser).

The completeness of genome assembly was evaluated by Benchmarking Universal Single Copy Orthologs (BUSCO, v5.0.0) [34] using the eudicots_odb10 database. The N50 values of contigs were used to evaluate the continuity of the genome. The accuracy of assembly was evaluated by the mapping rate and genome coverage of Illumina and PacBio reads using SAMtools (v1.13) [36]. The PacBio reads were aligned by minimap2 (v2.17) [73] and Illumina reads were aligned by BWA (v0.7.17) [35]. BCFtools (v1.13) was used to calculate the base accuracy of the assembly [37].

### Telomere and centromere identification

For telomere detection, plant telomeric sequences (TTTAGGG/CCCTAAA) were used to separately search the 100-kb regions at both ends of the chromosome. For centromere localization, Tandem Repeats Finder (TRF, v4.09) [74] was used to locate and display tandem repeat annotation with the recommended parameters: 2 7 7 80 10 50 500 -f -d -m.

### Genome comparison and synteny analysis

With a window length of 500 000 bp, the GC content, gene density, LTR content, TE content, tandem repeat content, SNP content, and InDel content of each window were counted and visualized using Circos software [75]. The WGS reads were mapped to the TSv2_T2T genome using default parameters with BWA­MEM (v0.7.17), and SAMtools (v1.13) and BCFtools (v1.13) were used for variant detection (SNPs and InDels).

Taking the length of 50 000 bp as the window, telomere existence, centromere length, LTR density, and collinearity of the TSv2_T2T and PN40024 genomes were calculated. Collinear block, PAV (presence–absence variation) analysis, and genome-wide sequence alignment were performed using MUMmer software [71]. The delta-filter −1 command was utilized to filter the comparisons. The show-coords -rcl command was used to obtain collinear blocks. The resulting collinear blocks were visualized using GenomeSyn [76]. Additionally, scanPAV (https://github.com/wtsi-hpag/scanPAV) was employed to analyze the PAV regions present in TSv2_T2T but absent in TSv1. The PAV density was calculated as the proportion of bases within the PAV coverage area.

The TSv2_T2T genome was compared with two previously published assembled genomes, *V. amurensis* (VA) [26] and *M. rotundifolia* cv. Noble (VR) [25], using minimap2 [73]. The comparison identified syntenic regions, structural rearrangements (duplications, translocations, and inversions), and sequence differences (deletions and insertions) using SyRI (v1.6) [77]. Plotsr (v0.5.4) [78] was used to generate visualizations of synteny and structural rearrangements among the three genomes.

### Genome annotation

The process of genome annotation is shown in Supplementary Data Fig. S8, and mainly included repetitive sequence annotation, gene structure prediction, and gene function prediction. Two strategies were used to annotate repetitive sequences. First, based on the repetitive sequence database RepBase (https://www.girinst.org/repbase/), RepeatMasker (v1.323) [79] and RepeatProteinMask were used to annotate homologous sequences. Second, RepeatModeler was used to establish the *de novo* repeat sequence library [80], RepeatMasker (v1.323) was used to predict the repeat sequences, and TRF (v4.09) [74] software was used to find the tandem repeat sequences in the genome. Three strategies were used to annotate the gene structure. First, BLAST (v2.2.28) [81] was used to compare the protein sequence of homologous species with the genome sequence of the new species to predict the gene structure. Second, Augustus (v3.3) [82], SNAP (v2006-07-28), and GeneMark (v4.33) were used for *de novo* prediction of the genome sequence data statistical features (such as codon frequency and exon–intron distribution). Finally, PASA (v2.10) and TopHat softwares were used to compare the Illumina sequencing data with the genome to help predict the gene structure. The gene sets predicted by the above three analyses were combined using EVidenceModeler (v1.11) [83]. The gene sets obtained by gene structure annotation were compared with a suite of protein databases for gene function annotation, including the NCBI nr, KOG/COG, Swiss-Prot (http://www.ebi.ac.uk/interpro/), KEGG (https://www.genome.jp/kegg/), GO (http://geneontology.org/page/go-database), TrEMBL, and Pfam (http://pfam.xfam.org/) databases. The known non-coding RNA library Rfam (https://rfam.org/) was used to compare and annotate rRNAs, snRNAs, and miRNAs. Prediction of tRNA sequences in the genome was done using tRNAscan-SE (v2.0) [84].

### Comparative genomic analysis

The genome sequence file (FASTA format) and genome annotation file (GFF format) of the species used for analysis were preprocessed to obtain the longest transcript of each gene, and then miscoded genes and genes exhibiting premature termination were filtered. Orthofinder (v2.5.4) [39] software for gene family analysis was used to obtain orthologous genes, paralogous genes, and single-copy homologous genes. A phylogenetic tree was constructed using single-copy orthologous genes and the divergence time was estimated. The construction of the species phylogenetic tree was performed in two main steps. First, MUSCLE (v3.8.31) [85] software was used to perform multiple sequence alignment of the protein sequences based on single-copy orthologous genes to obtain the protein-MSA data set, and the PAL2NAL (v14) [86] tool was used to obtain the CDS-MSA data set. Second, based on the protein-MSA and CDS-MSA data sets, the GTRGAMMA substitution model of RAxML (v8.2.10) [87] was used for phylogenetic tree construction with 1000 bootstrap replicates. MCMCTree in the software PAML (v4.8) [88] package was used to estimate the differentiation time point of each species. The CDS-MSA generated in the above phylogenetic tree analysis and the fossil calibration time as queried on the website TimeTree (http://www.timetree.org/) were used to construct the phylogenetic tree, and the software ggtree (v3.2.1) [89] was used for visualization. CAFE (v4.2.1) [90] software was used for gene family contraction and expansion analysis, and clusterProfiler (v4.0.5) [40] was used for GO enrichment analysis.

### Identification of NLR family genes

NLR-Annotator (https://github.com/steuernb/NLR-Annotator) was first used to identify the genomic segments of NLR genes for TSv2_T2T, VA, and VR. In addition, DRAGO2 (https://github.com/sequentiabiotech/DRAGO2-API) was used with default settings to annotate the NLR genes in the three genomes. All these results were merged after removing redundancy to obtain a comprehensive set of NLR loci. To predict the gene models of NLRs, training sets from AUGUSTUS [82] and SNAP, transcripts from RNA-seq, homologous proteins of *Arabidopsis* from PRGdb (http://prgdb.org/prgdb4/) and grape from ANNA (http://compbio.nju.edu.cn/app/ANNA/) were used for homologous annotation.

### Transcriptome analysis

Tissue-cultured seedlings were subjected to a hardening process and subsequently cultivated in a light incubator under controlled conditions (25°C, 16-h light/8-h dark cycle) for a period of 3 months. For the powdery mildew inoculation test, six plants were selected for each biological repetition. Inoculation was performed by applying powdery mildew spores to the leaves at nodes 3 and 4 according to previous studies [32, 45]. The transcriptome was sequenced using the Illumina platform following the method described in a previous study [91]. Briefly, the raw data were processed using fastp (v0.21.0) [92] to filter and trim low-quality reads. The clean reads were then aligned to the TSv2_T2T genome using HISAT2 [93]. Gene expression analysis was performed using StringTie and Ballgown. Enrichment analysis and visualization were conducted using the R package clusterProfiler. PlantCARE [94] and TBtools [95] were used for promoter element analysis and visualization.

## Supplementary Material

Web_Material_uhad260

## Data Availability

The raw sequencing data for the PacBio HiFi reads, ONT ultra-long reads, Hi-C reads, and Illumina reads have been deposited in the China National GeneBank DataBase (CNP0004225, https://db.cngb.org/).The RNA-seq data have been submitted to the NCBI Sequence Read Archive under BioProject ID PRJNA559985.
