# Supplementary material for: Telomere-to-telomere and gap-free genome assembly of a susceptible grapevine species (Thompson Seedless) to facilitate grape functional genomics
Source: Hortic Res. 2023 Dec 13;11(1):uhad260. doi: 10.1093/hr/uhad260 (PMC10822838; doi:10.1093/hr/uhad260)
Supplement: Web_Material_uhad260 [file web_material_uhad260.zip › Supplemental_Figure 23.9.4R1.docx]

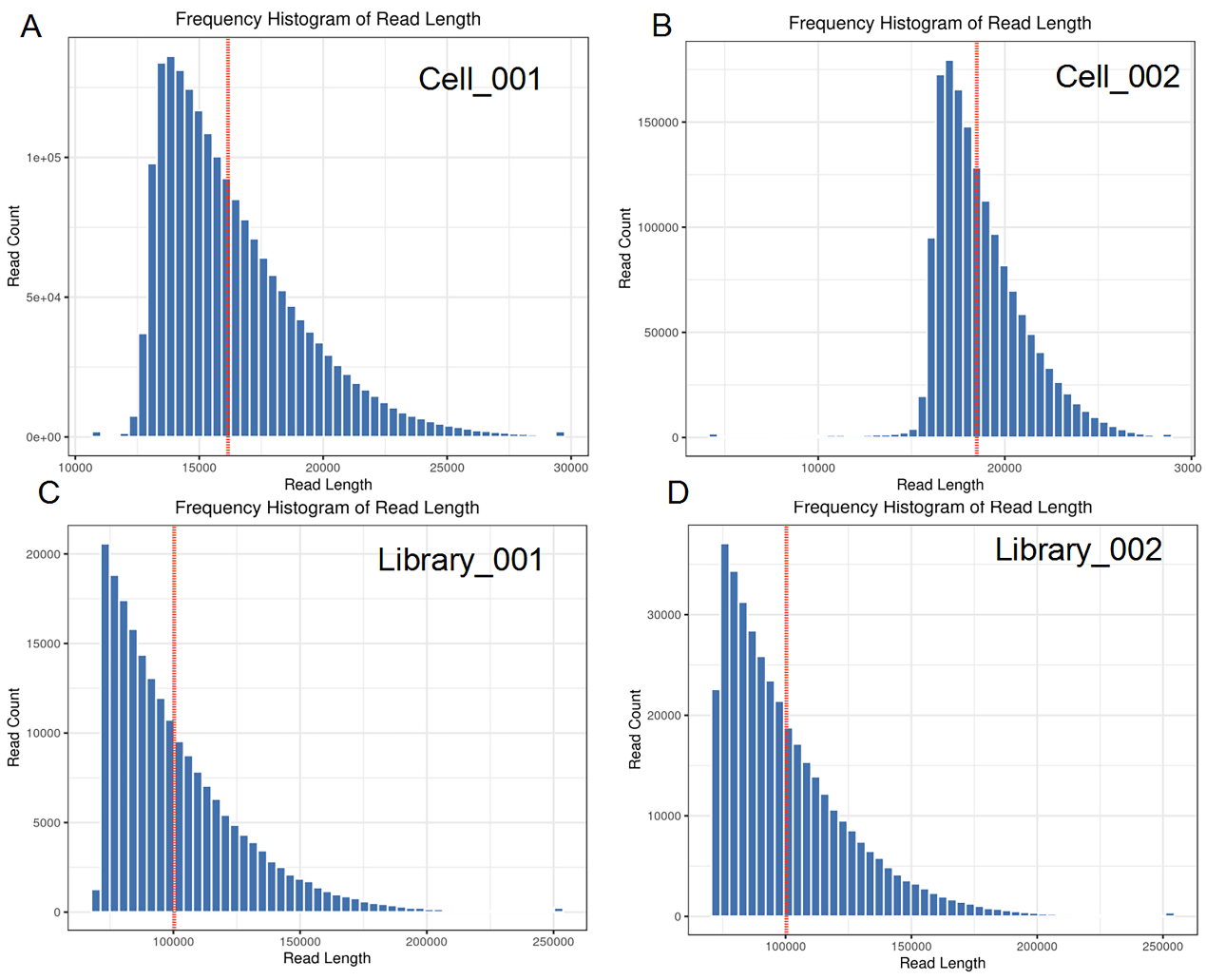


**Fig. S1**. Reading length distribution map of sequencing data.


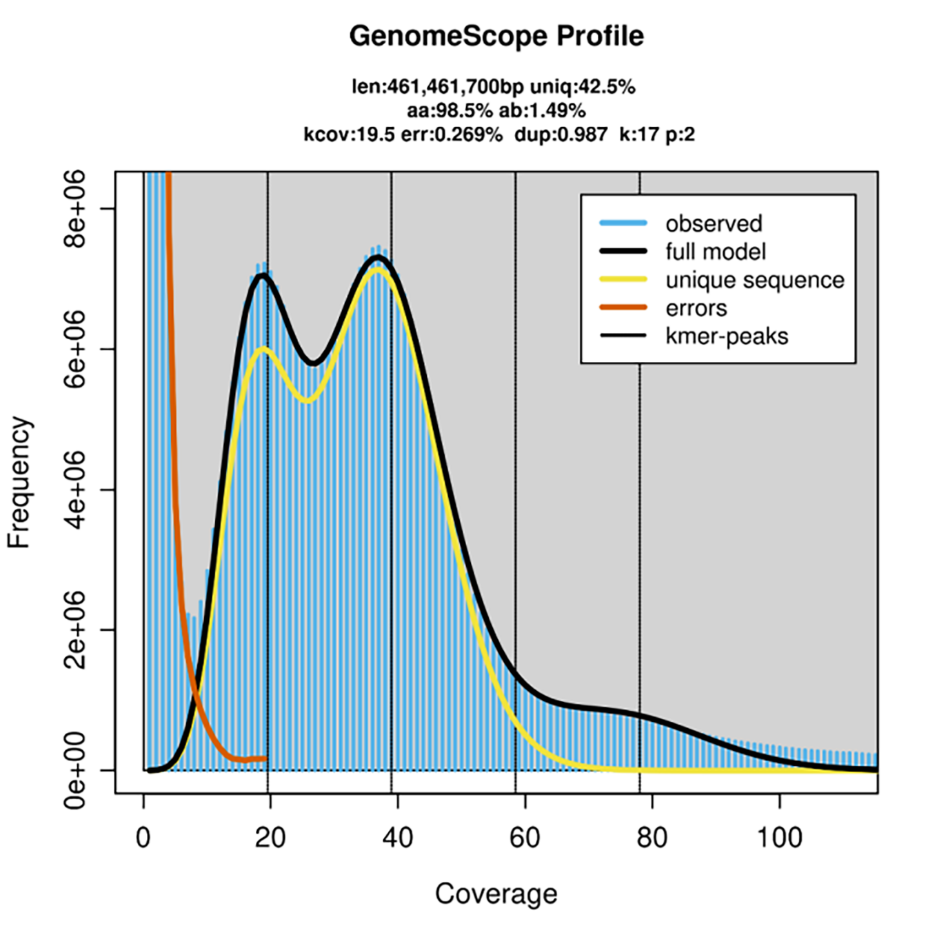


**Fig. S2**. Statistical analysis of genomic heterozygosity.


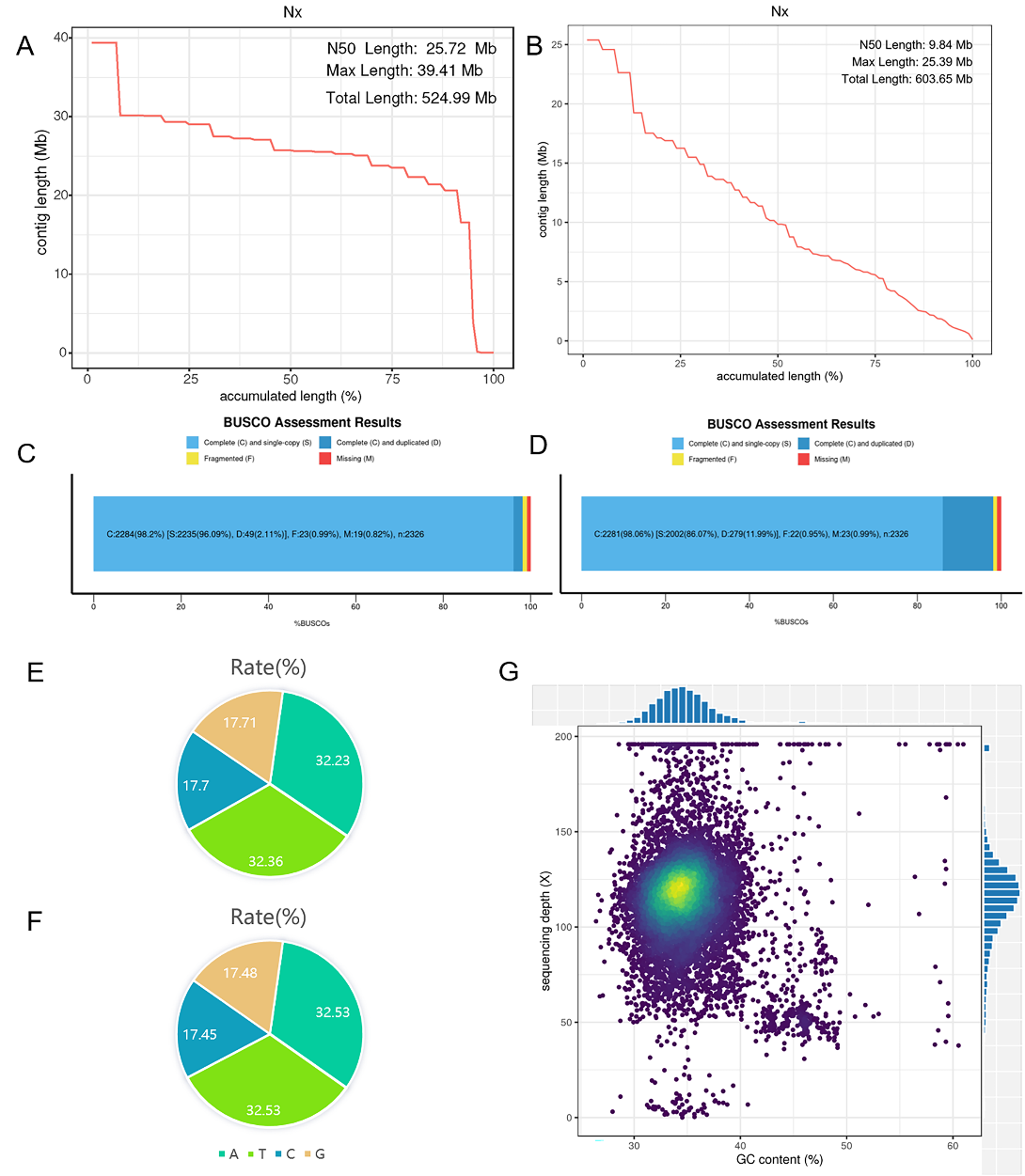


**Fig. S3**. Statistical analysis of genome assembly results.


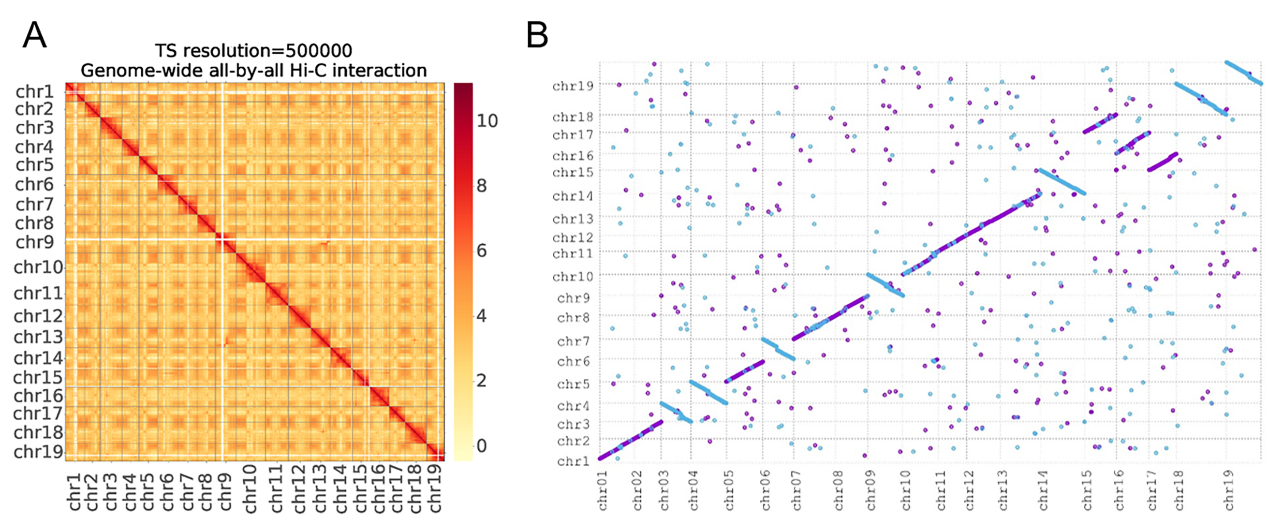


**Fig. S4.** The heatmap for Hi-C assisted assembly. The white rows and columns indicate bins with no valid interaction data.


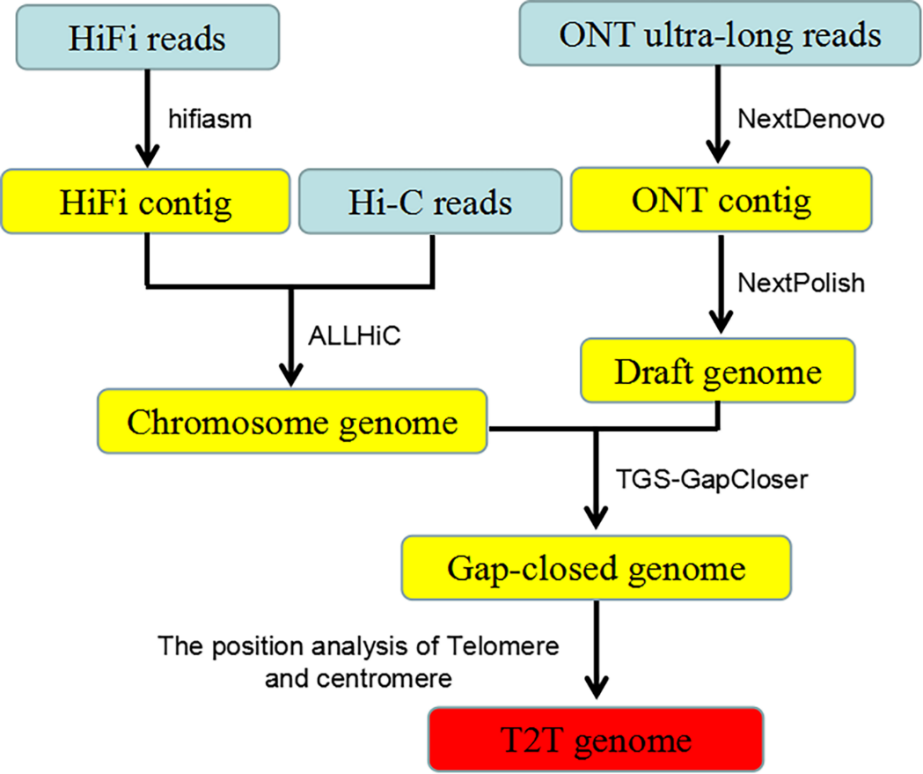


**Fig. S5**. Schematic diagram of T2T genome assembly.


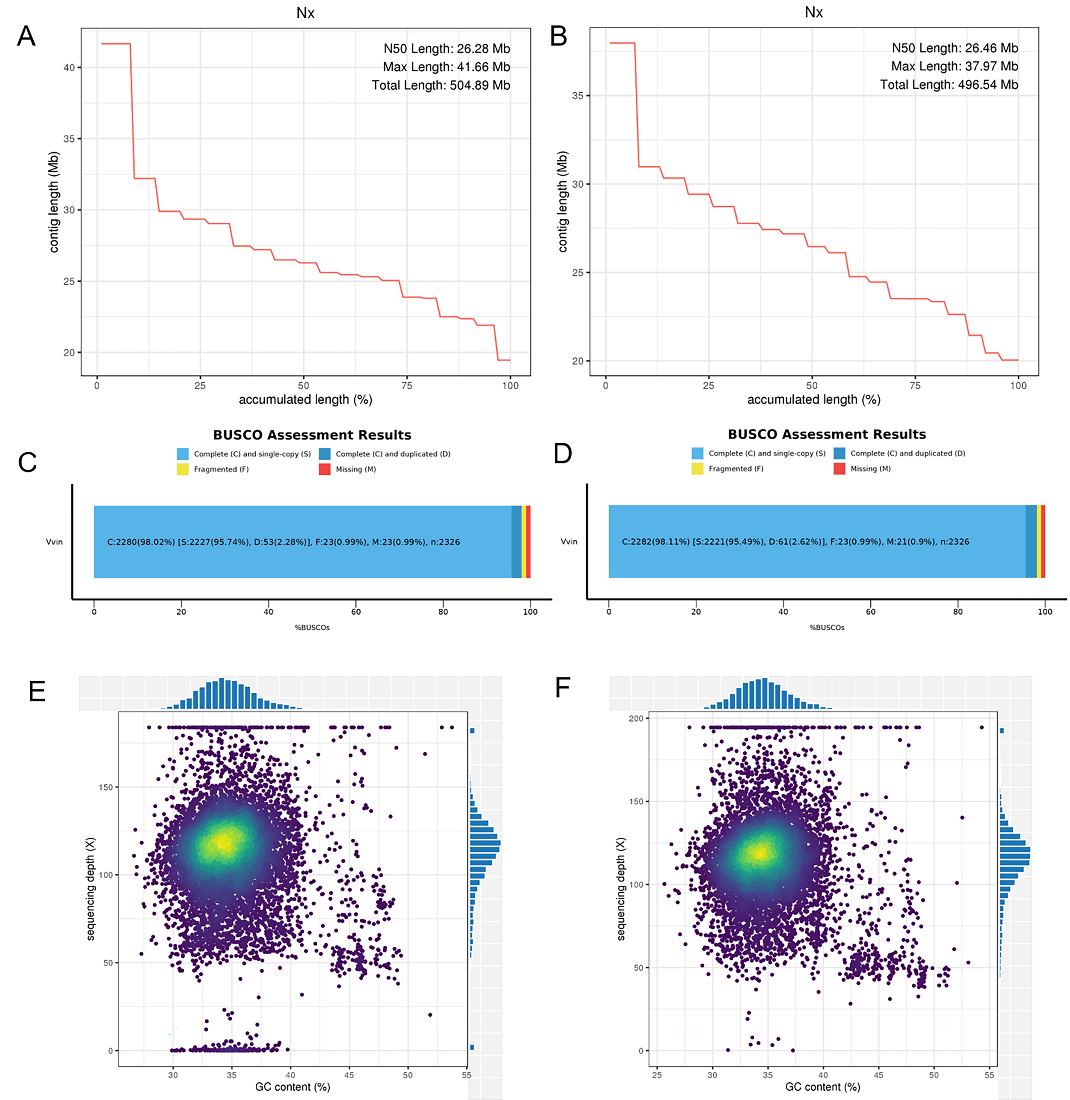


**Fig. S6**. Statistical analysis of Hap1_T2T and Hap2_T2T assembly results.


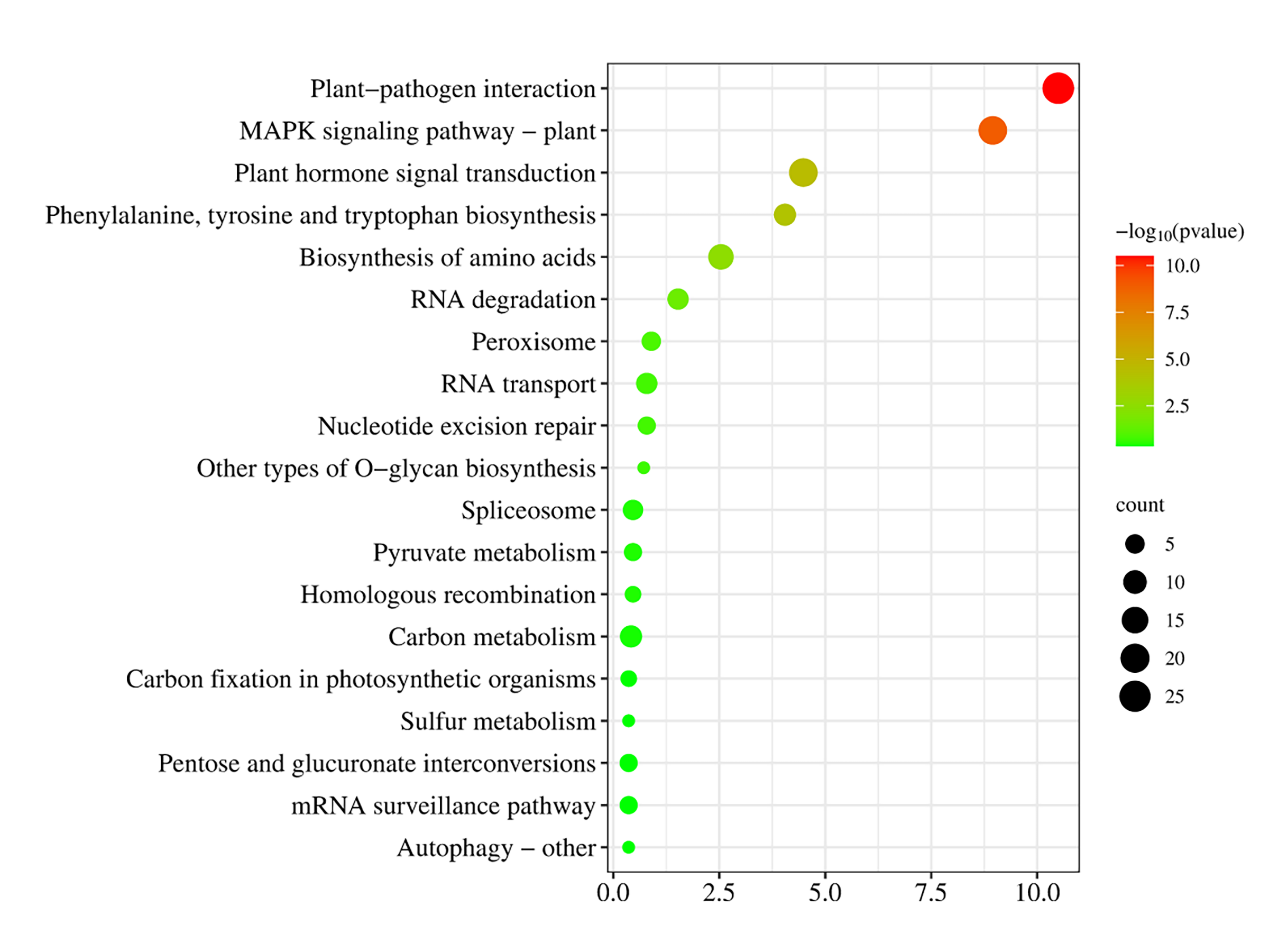


**Fig. S7**. Functional annotation of genes in the centromere region.


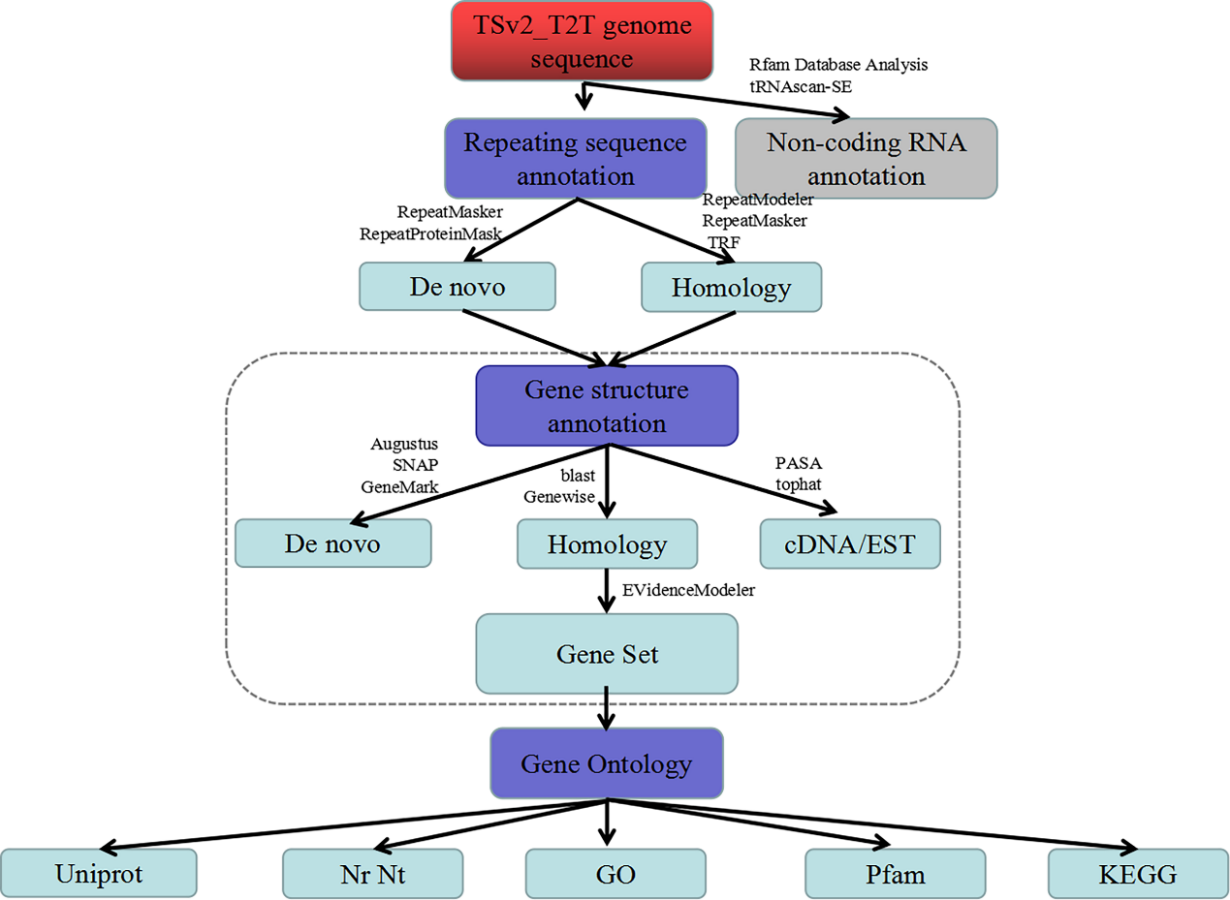


**Fig. S8**. Schematic diagram of gene annotation.


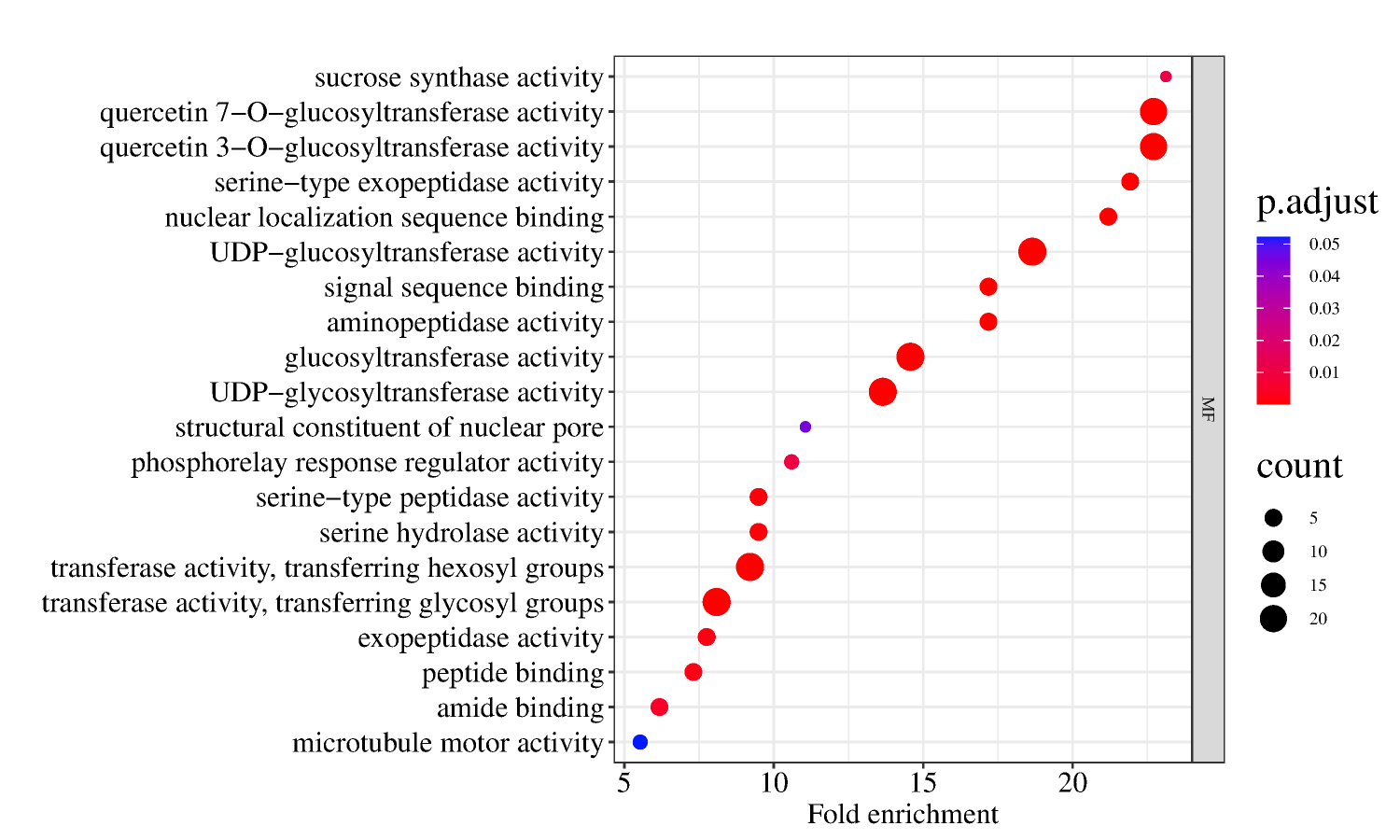


**Fig. S9**. Functional enrichment analysis of family expansion genes.


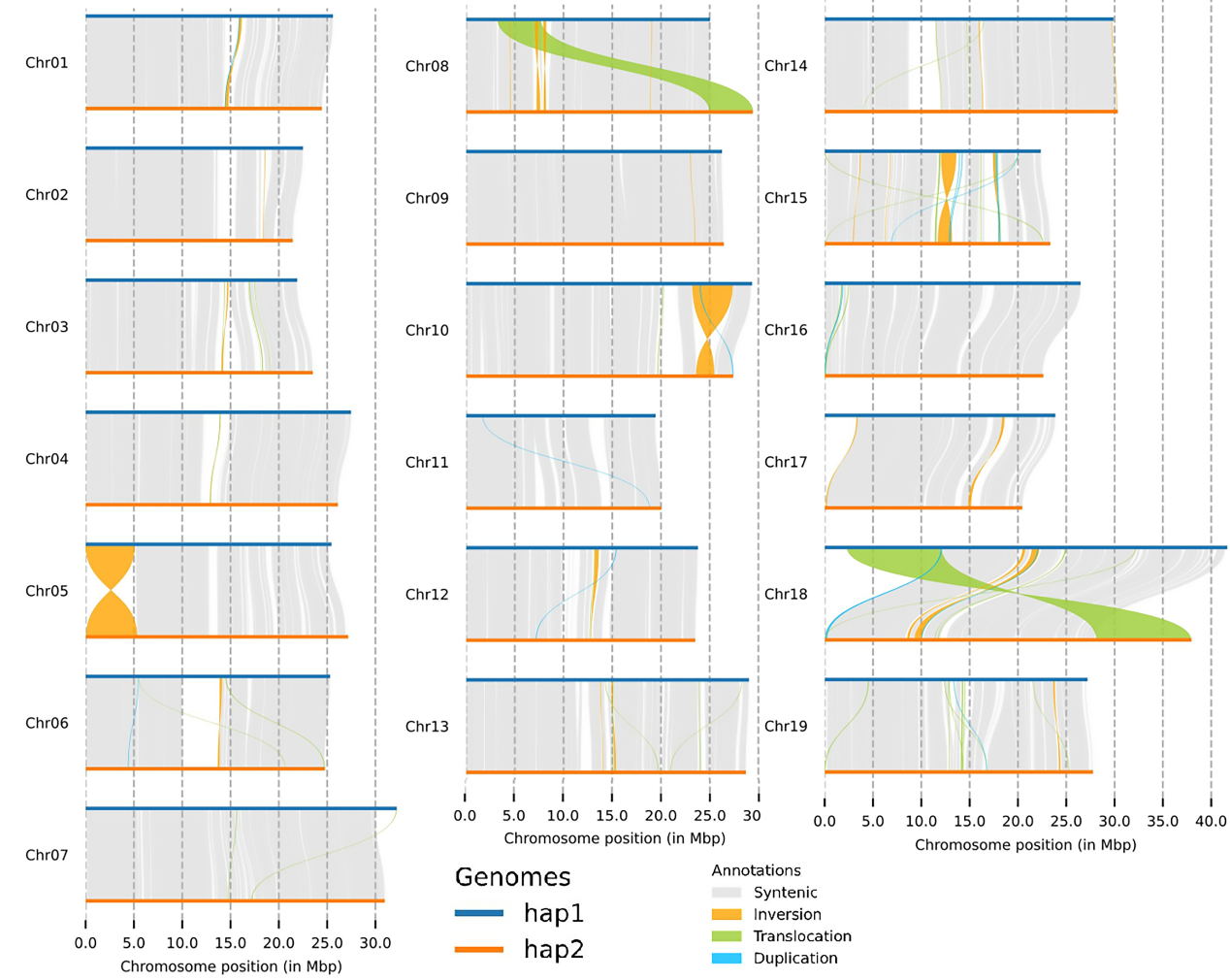


**Fig. S10**. Chromosome structural variation of Hap1 and Hap2.


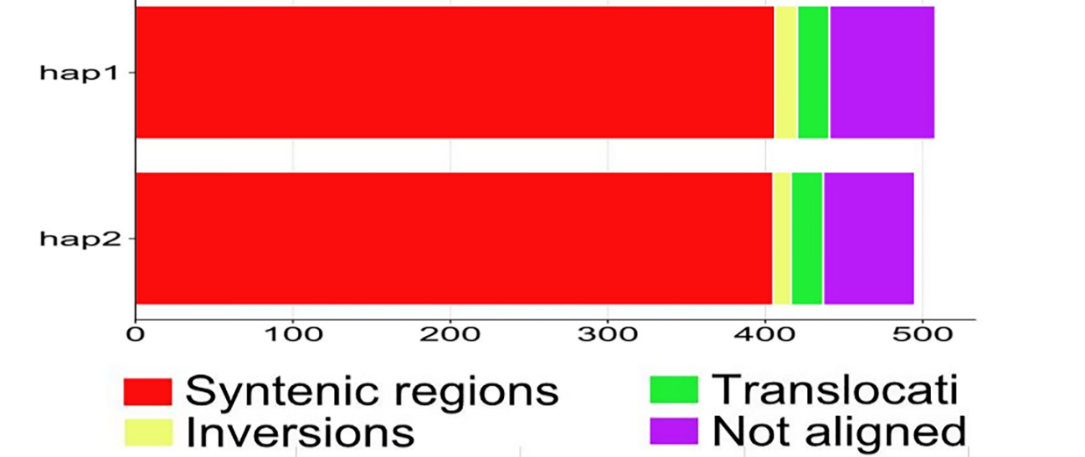


**Fig. S11**. Statistical structure of chromosome collinearity and variation.


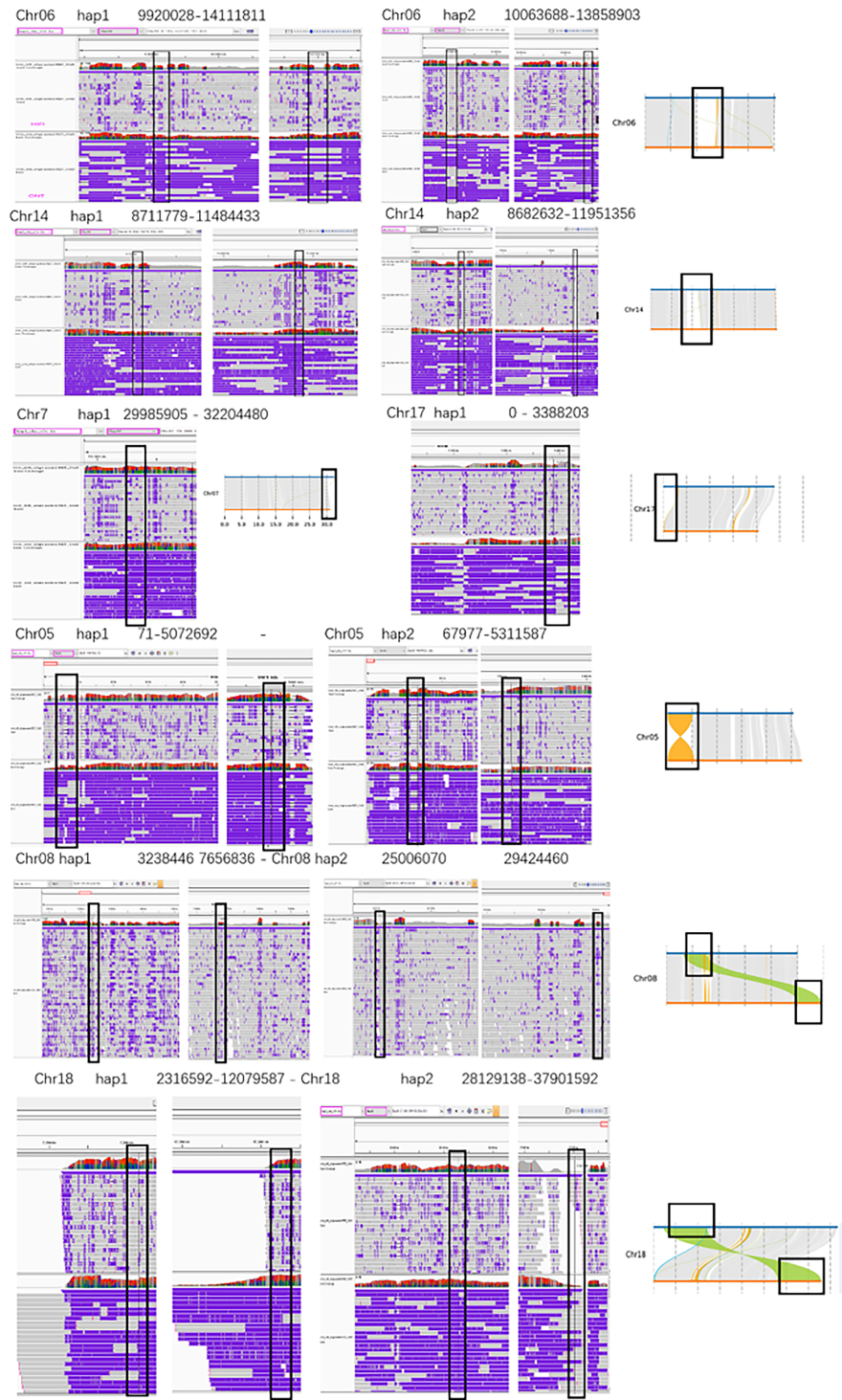


**Fig. S12**. Reads coverage statistics of structural variation.

**
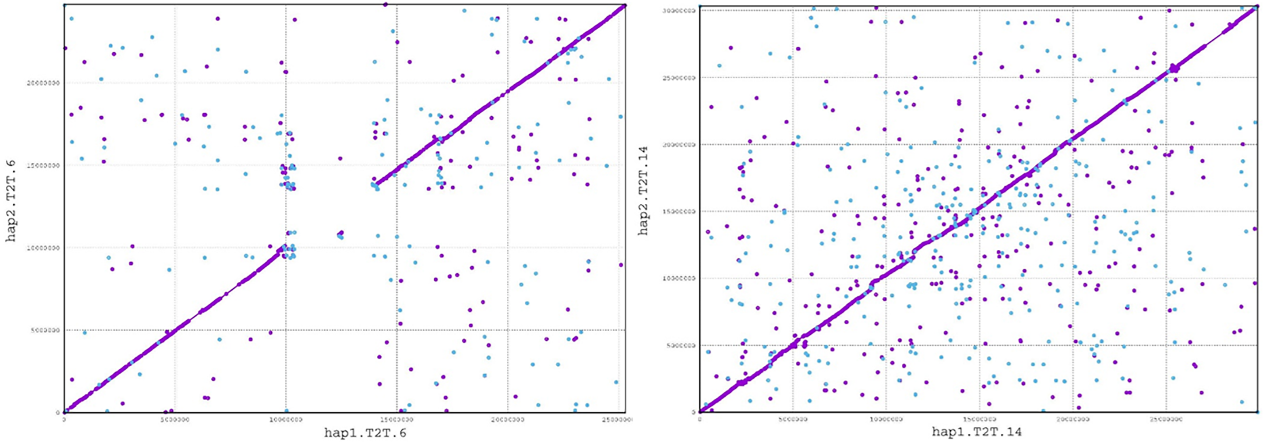
Fig. S13**. Collinearity analysis of unalignable regions of Chr6 and Chr14. Mummer4 is used for collinearity analysis.


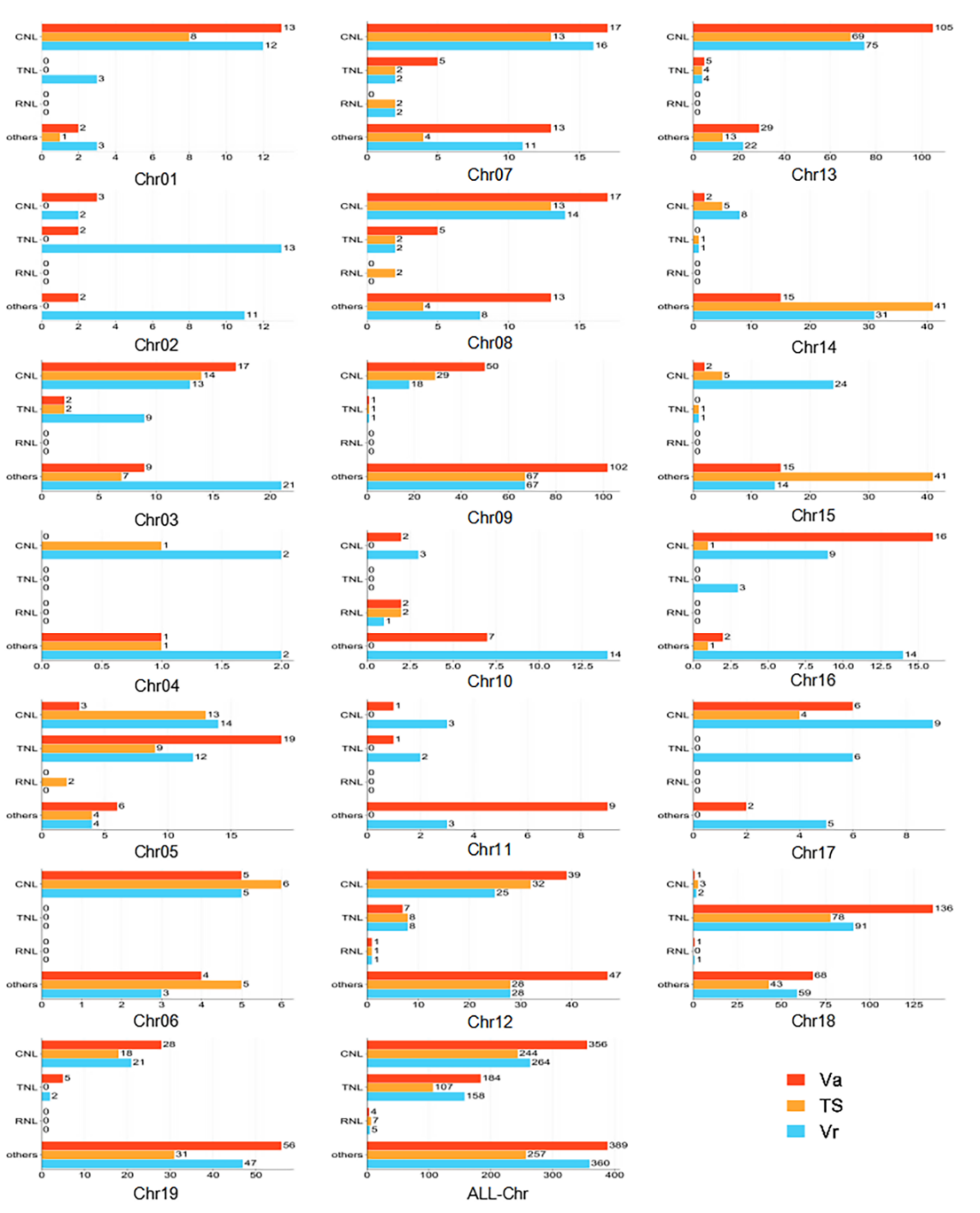


**Fig. S14**. Statistical analysis of the number of NLRs genes.

**
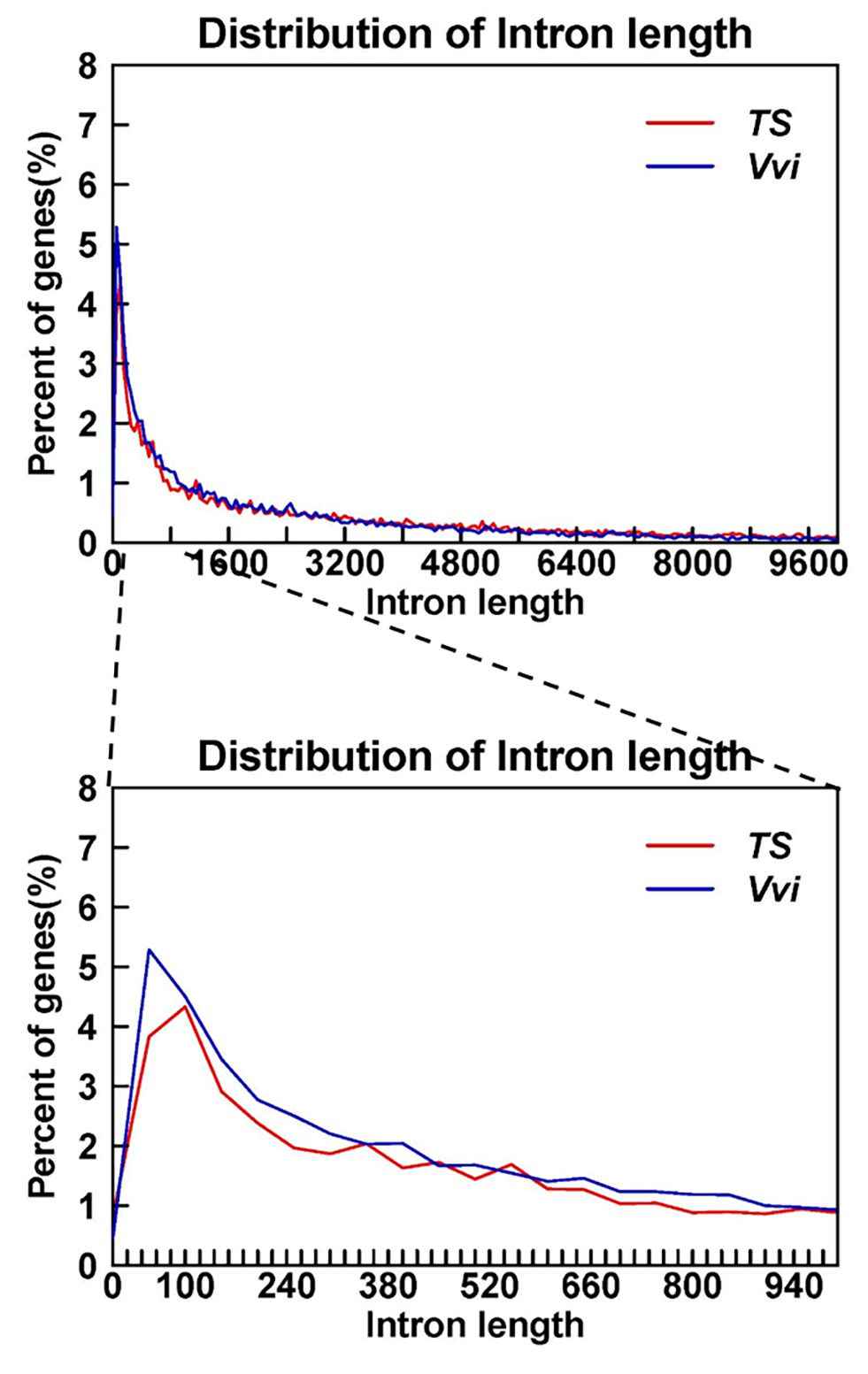
**

**Fig. S15**. The intron size of TSv2_T2T and PN40024v2.
